# Designing an implementation strategy to improve referral from general practice to a National Diabetes Prevention Programme using a Delphi survey with healthcare professionals and the Behaviour Change Wheel

**DOI:** 10.1136/bmjopen-2025-104300

**Published:** 2026-02-16

**Authors:** Clair Haseldine, Grainne O’Donoghue, Patricia M Kearney, Fiona Riordan, Margaret Humphreys, Liz Kirby, Sheena M Mc Hugh

**Affiliations:** 1 School of Public Health, University College Cork, Cork, Ireland; 2 University College Dublin, Dublin, Ireland; 3 Health Service Executive, Cork, Ireland

**Keywords:** Delphi Technique, Preventive Health Services, Implementation Science

## Abstract

**Abstract:**

**Objectives:**

While diabetes prevention programmes (DPPs) effectively reduce the risk of type 2 diabetes, optimising referral to these programmes is challenging. Our prior research (a qualitative study on the pilot of the National Diabetes Prevention Programme (NDPP) and a systematic review) identified a range of barriers and facilitators to referral from healthcare workers’ perspectives. This study aims to gain consensus on the main factors influencing referral to a newly established NDPP and using the Behaviour Change Wheel (BCW) to select behaviour change techniques (BCTs) for an implementation strategy to improve referral to the programme in the future.

**Design:**

A two-round modified online Delphi survey prioritised 17 barriers and facilitators of the referral process, followed by a mapping exercise with the BCW, which guided the identification of techniques to change referral behaviour from general practice.

**Setting:**

The survey took place online with healthcare professionals working in the primary care setting in Ireland (April to June 2024). The NDPP was in the pilot phase and was not available in all areas. This study sought to learn from this pilot phase to inform the referral process, which was not yet fully established.

**Participants:**

Healthcare professionals eligible to refer or involved in referral to the NDPP in Ireland (general practitioners, practice nurses and dietitians delivering the NDPP) took part in the Delphi survey. Recruitment was through a number of gatekeepers, a health service manager and professional groups who shared invitations to participate with eligible healthcare professionals.

**Outcome measures:**

In the Delphi survey round 1, respondents were asked to rate the importance of 17 factors (nine facilitators and eight barriers) influencing referral on a 5-point Likert scale (not important to very high importance) and an open text box captured other suggested important factors. Barriers included limited practical information about the availability of the programme, concerns about workload, competing priorities and concern about patient motivation, the time commitment for patients and referral delays. Facilitators included electronic referral and feedback, promotion of the programme by healthcare professionals and consultation with patients before referral. Consensus was defined as agreement of ≥70% for each factor in the combined categories of high importance/very high importance, low/moderate importance or not important. Factors not reaching consensus after the first round were included in round 2 with any new factors from round 1. Factors that did not reach consensus or reached consensus as not important or of low/moderate importance were excluded. Only factors reaching consensus as being of high importance/very high importance across the two rounds were included in the final prioritised list.

**Results:**

The Delphi survey had 37 responses to round 1 and 23 (62%) responses to round 2. 12 factors reached consensus as being of high/very high importance to improve referral. The 12 factors are mapped to seven intervention functions in the BCW and to nine key BCTs (feedback on the outcome of the behaviour, social support, instruction on how to perform a behaviour, information about the health consequences, information about social and environmental consequences, demonstration of the behaviour, prompts/cues, credible source and restructuring the physical environment). The strategy to improve referrals should include education delivered by educators to referrers, educational materials on the DPP and practical support to facilitate referrals. The health service should continue to provide electronic referrals and electronic prompts to refer could be considered as part of the electronic health record.

**Conclusion:**

This study systematically prioritises factors perceived to influence referral and identifies BCTs to improve referral to an NDPP. The BCTs are a starting point for a strategy to improve referral to DPPs. Further consultation with stakeholders is recommended to discuss the acceptability, feasibility and operationalisation of the BCTs in the Irish setting.

**Strengths and limitations of this study**

- The study phases and methods align with the development phase of the UK Medical Research Council’s updated guidance for developing complex interventions, which includes involving stakeholders, having a development team with expertise, reviewing existing literature, including primary data, and consideration of the context.
- This study used the Behaviour Change Wheel, a systematic and structured method to develop a strategy to improve referral to a National Diabetes Prevention Programme.
- This study uses a Delphi survey, a widely used method in healthcare research, within the context of the Behaviour Change Wheel approach to gain consensus and prioritise the main barriers and facilitators influencing referral to the National Diabetes Prevention Programme in Ireland.
- The Delphi survey did not capture context-dependent factors, which could influence referral, such as the practice characteristics of the healthcare professionals surveyed.

## Introduction

 Type 2 diabetes is a growing global health problem which causes considerable morbidity and mortality.^1^ The global prevalence of adults (20–70 years) living with diabetes was estimated to be over 10.5% (536.6 million people) in 2021.^2^ An estimated 90% of cases are type 2 diabetes, with obesity, sedentary lifestyles and ageing populations contributing to the rising prevalence.^3^ The prevalence is predicted to continue rising, leading to calls for urgent preventative public health measures to be taken.^3^

Diabetes prevention programmes (DPPs) are effective at preventing type 2 diabetes in people who are at high risk of developing the disease.^4^ DPPs are intensive and structured behaviour change interventions that encourage healthy eating and physical activity. In response to the growing prevalence of type 2 diabetes and the evidence of effectiveness, countries around the world have begun to implement national and regional programmes,^4^ with primary care playing an important role in identifying and referring those at risk.^5^ The full potential of prevention programmes cannot be realised however, if there are low rates of referral and uptake to the programmes.^6 7^

In a cross-sectional survey in the USA, only 4.2% of a nationally representative sample of adults eligible to participate were referred.^6^ A cohort study in England using electronic health record data found that 14% of people identified with pre-diabetes had a code in their record indicating that they had been referred to the National Health Service (NHS) DPP.^8^ Another study in England found referrals to the NHS DPP varied substantially between primary care practices, with higher rates of referral from practices with higher quality indicators (one SD higher level of achievement on the quality score was associated with an 11% higher referral rate).^7^ In 2023, Teoh *et al* conducted a quantitative systematic review of knowledge, attitudes and practices of pre-diabetes among healthcare professionals and people with pre-diabetes.^9^ Eight of the 21 studies included in the review assessed if healthcare professionals would consider referring their patients to DPPs and found that less than 36% would consider it. The low referral rates and healthcare professionals’ reluctance to refer to DPPs warrant further investigation.

Despite the evidence of low referral to DPPs, to date, few strategies have been designed to improve referrals. Chambers *et al* combined a modification to the electronic health record with education for healthcare professionals to increase referrals to DPPs in six health centres in New York City.^10^ While the strategy was found to significantly increase referrals, the number of eligible people referred was extremely low, at an average of one per month before the strategy to six per month after the strategy (total eligible population not reported). The authors do not describe any systematic process to inform the development of the strategy. Keck *et al* conducted a cluster randomised controlled trial to investigate the effect of a strategy including education, electronic health record identification of people with pre-diabetes and a pre-diabetes clinician champion.^11^ The healthcare professionals who received the strategy referred 6.9% of people with pre-diabetes to DPPs compared with 1.5% of those who did not. The strategy was informed by systematic processes using formative research. Holliday *et al* piloted a toolkit which contained algorithms to retrospectively identify people with pre-diabetes from electronic health records or sample patient flow handouts with instructions for opportunistic screening and referral to DPPs using the RE-AIM (Reach, Effectiveness, Adoption, Implementation, Maintenance) implementation science framework.^12^ All 26 primary care practices and health systems in the pilot increased referrals, from no referrals at baseline to 5460 during the pilot intervention. Providing financial incentives to referrers has had mixed results. McManus *et al* investigated the effect of financial incentives on general practice and found that incentives linked to the number of referrals to the DPP (outcome incentives) resulted in 84% more referrals than practices that did not receive incentives.^13^ Providing structural (eg, payment based on practice size) or process incentives (eg, payment for identifying eligible participants) did not result in an increase in referrals.

The health service in Ireland is implementing a National Diabetes Prevention Programme (NDPP). The programme was piloted in 2021/2022 as an online programme and it is currently being rolled out nationally on a phased basis, with face-to-face delivery (since mid-2024) in addition to online delivery. The NDPP is accessed via general practitioner (GP) referral to community specialist teams outside of the hospital setting. Prior research, including a systematic review exploring healthcare workers’ perspectives on referral to DPPs^5^ and a qualitative study among attenders and educators in the pilot of the NDPP in Ireland,^14^ identified multiple barriers and facilitators to referral. These barriers and facilitators include healthcare workers’ knowledge of the effectiveness and availability of programmes, the clarity and ease of use of the referral pathway, staff and resources for referral and concern that attending the programme would unduly burden (time and financial) their patients.^5^ It is important to prioritise the factors, as the list may be so long that not all factors could feasibly be included in a strategy and considering factors that affect referral during early implementation can identify modifiable targets to improve referral rates in the future.

This study aims to gain consensus on and prioritise the most important factors influencing referral to an NDPP among healthcare professionals involved in referral. The study also aims to use the Behaviour Change Wheel (BCW) to select behaviour change techniques (BCTs) for an implementation strategy to improve referral to the programme in the future. BCTs are part of the systematic approach to designing strategies. They are the observable and reproducible techniques included in the strategy to change behaviour.^15^

## Materials and methods

### Overarching design

This implementation study, aiming to develop a theoretically informed strategy to improve referral, is broadly based on the BCW.^16^ The BCW is a systematic method to guide the development of strategies to change behaviour. It was developed by synthesising 19 frameworks of behaviour change. The BCW outlines a three-stage process to develop a behaviour change intervention from identifying and understanding the behaviour to target, to identifying the BCTs with the potential to change the behaviour. The final part of stage three of the BCW involves identifying a mode of delivery for the intervention, which was not addressed in this study.

**Figure 1:**
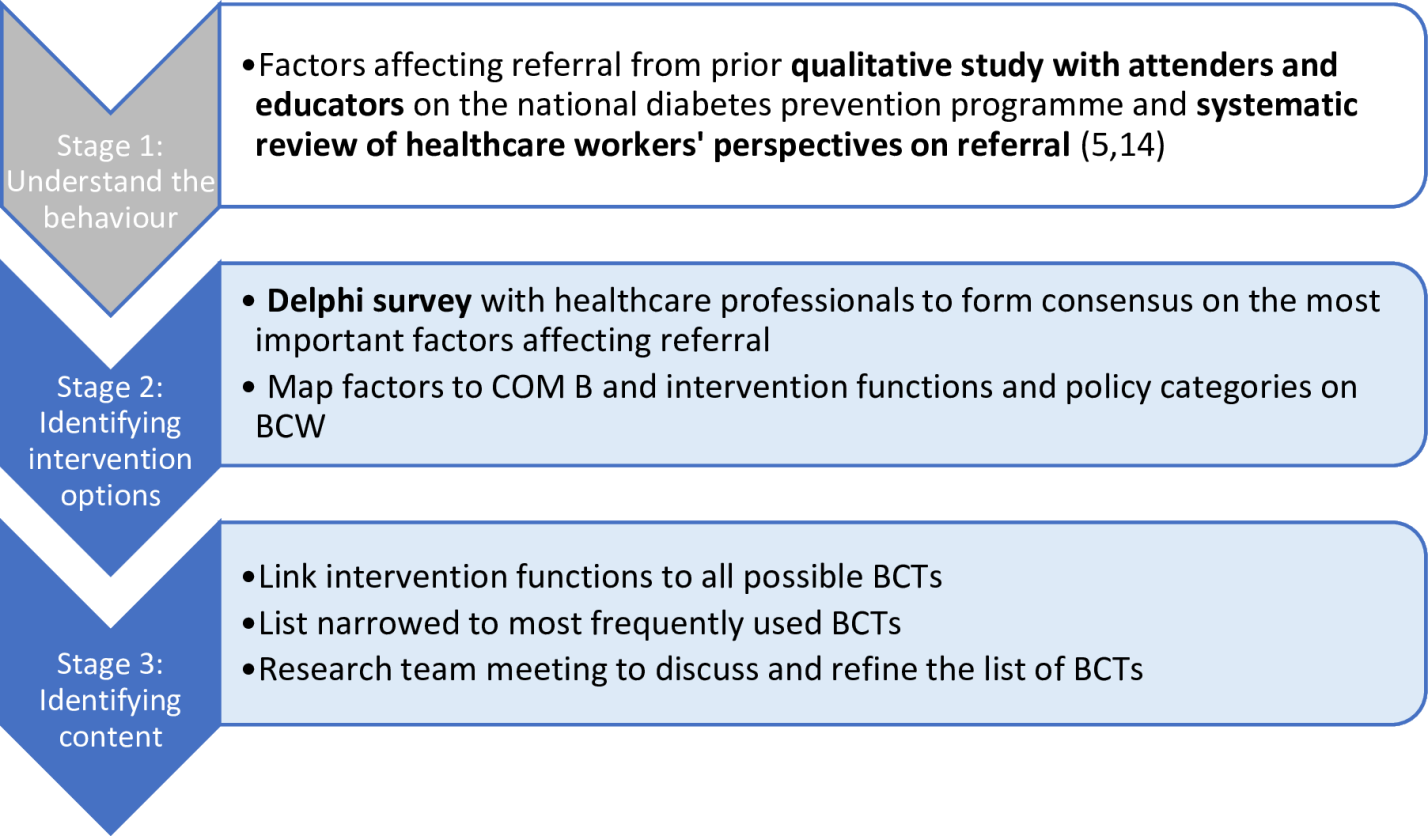
Stages of the BCW applied to development strategy and research processes/methods used.^16^ BCTs, behaviour change techniques; BCW, Behaviour Change Wheel; COM-B, Capability, Opportunity, Motivation, Behaviour.

This study focuses on stages 2 and the initial part of stage 3 of the BCW, namely *identifying intervention options* and *identifying content*. Stage 1 of the BCW, which aims to *understand the behaviour*, was addressed in prior research using the Theoretical Domains Framework (TDF) to provide a detailed analysis of referral behaviour (5,14). See figure 1 for an outline of the study linked to the stages of the BCW.

### Patient and public involvement

A patient and public involvement group was involved in the larger research project, which included this study^14^ but they did not contribute to this study.

### Delphi study design (BCW stage 2)

This study used a two-round online modified Delphi to prioritise factors influencing referral to the NDPP. Delphi surveys use anonymous, iterative rounds with controlled feedback to gain consensus.^17^ The modified Delphi differs from the traditional Delphi in that the researchers, and not the survey participants, generate the list of factors under investigation in round 1, either from a previous literature review or studies.^18^

#### Context

At the time of the Delphi survey (April to June 2024), the NDPP was in the pilot phase. It was not yet available in all areas, and a formal referral process had not yet been fully established. During the pilot, participants eligible for the NDPP were identified through existing waiting lists for primary care dietitians and by referral from GPs who had been informed of the programmes in their areas.

At the time of the Delphi study, electronic referral was available in some areas as part of a wider Structured Chronic Disease Management Programme in the Irish Health Service Executive. The Structured Chronic Disease Management Programme provides annual preventative GP and practice nurse visits for people at high risk of cardiovascular disease or type 2 diabetes (as well as people with asthma or chronic obstructive pulmonary disease).

#### Participants

Participants in the survey—referred to as Delphi panel members—consisted of healthcare professionals eligible to refer or involved in the referral process to the NDPP. GPs and practice nurses are involved in referral to the programme in Ireland. Educators on the NDPP (mainly community-based dietitians) are also involved as they contact potential referrers, informing them about the programme and contact people referred to take up a place on the programme.

#### Recruitment of panel members

We recruited panel members through a number of gatekeepers. The programme manager of the NDPP shared the invitation email with educators on the NDPP. The research team (experienced researchers in public health and a healthcare professional/researcher with experience of delivering diabetes prevention services in a community setting) invited healthcare professionals through professional groups (GP, practice nurse and diabetes special interest groups for healthcare professionals). These healthcare professionals may or may not have referred to the NDPP. The gatekeepers were encouraged to share the invitations with all eligible healthcare professionals, so the total number of invites sent out is not known. Studies have indicated that 10–15 respondents could be sufficient for consensus if their backgrounds are homogenous.^19^ We had a minimum target of 15 panel members to allow for drop-off between the rounds of the survey.

The healthcare professionals were invited by email, which included details of the study and a link to the online survey (Qualtrics). Healthcare professionals were instructed to complete the survey within 2 weeks of the start date. Only those who completed the first round received the second-round survey. Reminder emails were sent to complete each round. The two rounds took place from April to June 2024.

#### Survey development

A list of factors affecting referral was generated from a previous qualitative study with attenders of and educators of the NDPP^14^ and a systematic review of 37 studies on barriers and facilitators to referral from healthcare workers’ perspectives^5^ (online supplemental files 1 and 2). These studies informed the list, as the qualitative study^14^ was the only published article on a NDPP in Ireland at that time and the systematic review was a comprehensive analysis of the international literature on perspectives on referral to DPPs.^5^ These two studies reflect the first stage of the BCW framework, namely *understanding the behaviour*, and they sought to understand factors specific to the Irish context^14^ and broader international evidence.^5^ As such, the list of items reflects broader contextual factors that were relevant.

The research team and the NDPP management team (programme manager and a self-management support lead) discussed the factors identified in an iterative process with multiple rounds of discussions to ensure the survey was clear and relevant to healthcare professionals working in the Irish context. Factors relating to insurance cover were not included as there is no charge to attend the NDPP in Ireland. The Delphi survey was trialled with two healthcare professionals to ensure clarity and understanding. To maximise responses and reduce the response burden, each round of the survey was designed to be completed in 10 min or less.

### Delphi rounds

#### Delphi round 1

The survey collected demographic information: profession, gender, years qualified and email address to enable contact across rounds. Respondents were also asked whether they were aware of the NDPP and whether they had referred to the programme. The survey then asked respondents to rate the importance of 17 factors (nine facilitators and eight barriers) influencing referral on a 5-point Likert scale (not important to very high importance). There was an open text box to add comments after the rating and there was an open question to suggest new factors that they felt were important but were missing from the list presented.

Following the rating, respondents were asked to rank their top five most important factors. This ranking was included to provide a method to prioritise the most important factors when respondents rate all or many factors as important or very important.

#### Delphi round 2

In the second round, administered 2 weeks after the first survey, respondents were presented with group ratings of importance from round 1 and were reminded of their original scoring. Factors that reached consensus in round 1 were removed and respondents were asked to re-rate the remaining factors as well as any new factors from round 1. The respondents were asked to rank their top five from the list of remaining factors and new factors from round 1.

This resulted in a rating of factors that reached consensus across the two rounds of the survey and the potential to rank the top five factors from each round if necessary.

### Data analysis

While definitions of consensus in Delphi studies vary, a consensus of 70% or over is commonly used.^20^ This study defined consensus as 70% or over for the combined categories of high importance/very high importance, low/moderate importance or not important, similar to previous research using modified Delphi.^21^ Only factors reaching consensus as high/very high importance were included in the final prioritised list. Factors that did not reach consensus or reached consensus as not important or of low/moderate importance were excluded. Respondents were required to move through all of the sections of the survey and press the submit button to be analysed. The number of respondents who started the survey but did not move beyond the demographic section was recorded as incomplete.

Quantitative analysis was conducted in SPSS with descriptive statistics. Free text responses were collated to identify common topics which were discussed by the research team and included as factors in round 2 if agreed that they were new factors not already captured in round 1.

### Mapping to intervention functions (BCW stage 2)

The lead researcher (CH) mapped the factors prioritised in the Delphi survey (previously mapped to the TDF) to the categories in the Capability, Opportunity, Motivation, Behaviour (COM-B) to summarise the aspects of Capability (C), Opportunity (O) and/or Motivation (M) that were considered most important to address.^16^ The mapping process followed the guidance in the BCW manual.^16^ The TDF gives a detailed understanding of the specific factors influencing referral behaviour and the COM-B is a higher-level classification which facilitates the mapping of these factors to intervention functions to change that behaviour (online supplemental file 3). The COM-B categories were mapped to nine intervention functions within the BCW: Education, Persuasion, Incentivisation, Coercion, Training, Restriction, Environmental restructuring, Modelling and Enablement, which are broad categories identified as the most likely to bring about the changes. The research team discussed the identified intervention functions and eliminated any that were inappropriate in the context of general practice referral to the NDPP.

### Selecting BCTs (BCW stage 3)

In the BCW, each intervention function links to a long list of BCTs.^15 16^ To narrow this list, we used guidance from Abraham *et al* on the BCTs which are most frequently used within each function.^22^ The research team (CH, GO’D, PMK, FR, SMMcH) discussed the BCT shortlist in an online meeting (lasting 90 min) by considering how the BCT could be operationalised within the context of referral to the NDPP. Different approaches are used for selection of BCTs, sometimes led by researchers and other times involving health service stakeholders.^23 24^ We opted for a research-led approach to minimise the burden on healthcare professionals who were already heavily involved in the qualitative study to *understand the behaviour* (BCW stage 1) and the Delphi survey. The five members of the research team involved in this discussion were female academics, four of whom were healthcare professionals. The lead author (CH) was a PhD scholar/healthcare professional with an interest in chronic disease prevention and experience delivering a DPP. The results of the meeting were summarised by the lead author (CH) with the final list of BCTs returned to the research team to check for accuracy (see Consolidated criteria for Reporting Qualitative research checklist online supplemental file 4).

## Results

### Delphi survey results (BCW stage 2)

37 complete responses were received for round 1. A further 13 incomplete responses were recorded. In round 2, 23 (62%) complete responses were received and two were incomplete. Only completed responses were included in the analysis. The professional background and demographic data for the respondents are shown in table 1. There was representation from all of the professionals in both rounds of the survey, with experience ranging from 5 years or less to over 30 years. The majority (68%, n=25, round 1; 74%, n=17, round 2) were aware of the NDPP, though less than a quarter (24%, n=9, round 1; 22%, n=5, round 2) had referred.

**Table 1:** Demographic data for respondents rounds 1 and 2

	Round 1 n=37 n (%)	Round 2 n=23 n (%)
Profession		
General practitioner	10 (27)	7 (30)
Practice nurse	20 (54)	11 (49)
Dietitian/educator	7 (19)	4 (17)
Missing	0 (0)	1 (4)
Years qualified		
≤5	5 (14)	1 (4)
6–15	9 (24)	5 (22)
16–30	16 (43)	10 (44)
>30	7 (19)	6 (26)
Missing	0 (0)	1 (4)
Gender		
Man	6 (16)	4 (17)
Woman	30 (81)	17 (74)
Prefer not to say	1 (3)	1 (4)
Missing	0 (0)	1 (4)
Aware of the National Diabetes Prevention Programme		
Yes	25 (68)	17 (74)
General practitioner	4 (11)	4 (17)
Practice nurse	14 (38)	9 (39)
Dietitian/educator	7 (19)	4 (17)
No	12 (32)	5 (22)
General practitioner	6 (16)	3 (13)
Practice nurse	6 (16)	2 (9)
Dietitian/educator	0 (0)	0 (0)
Missing	0 (0)	1 (4)
If yes, how they heard about the National Diabetes Prevention Programme*		
Work	9 (24)	
Professional body	3 (8)	
Training	6 (16)	
Missing	9 (24)	
Referred to the National Diabetes Prevention Programme		
Yes	9 (24)	5 (22)
General practitioner	2 (5)	2 (9)
Practice nurse	5 (14)	1 (4)
Dietitian/educator	2 (5)	2 (9)
No	28 (76)	17 (74)
General practitioner	8 (22)	5 (22)
Practice nurse	15 (41)	10 (44)
Dietitian/educator	5 (14)	2 (9)
Missing	0 (0)	1 (4)

* More than one response possible.

**Figure 2:**
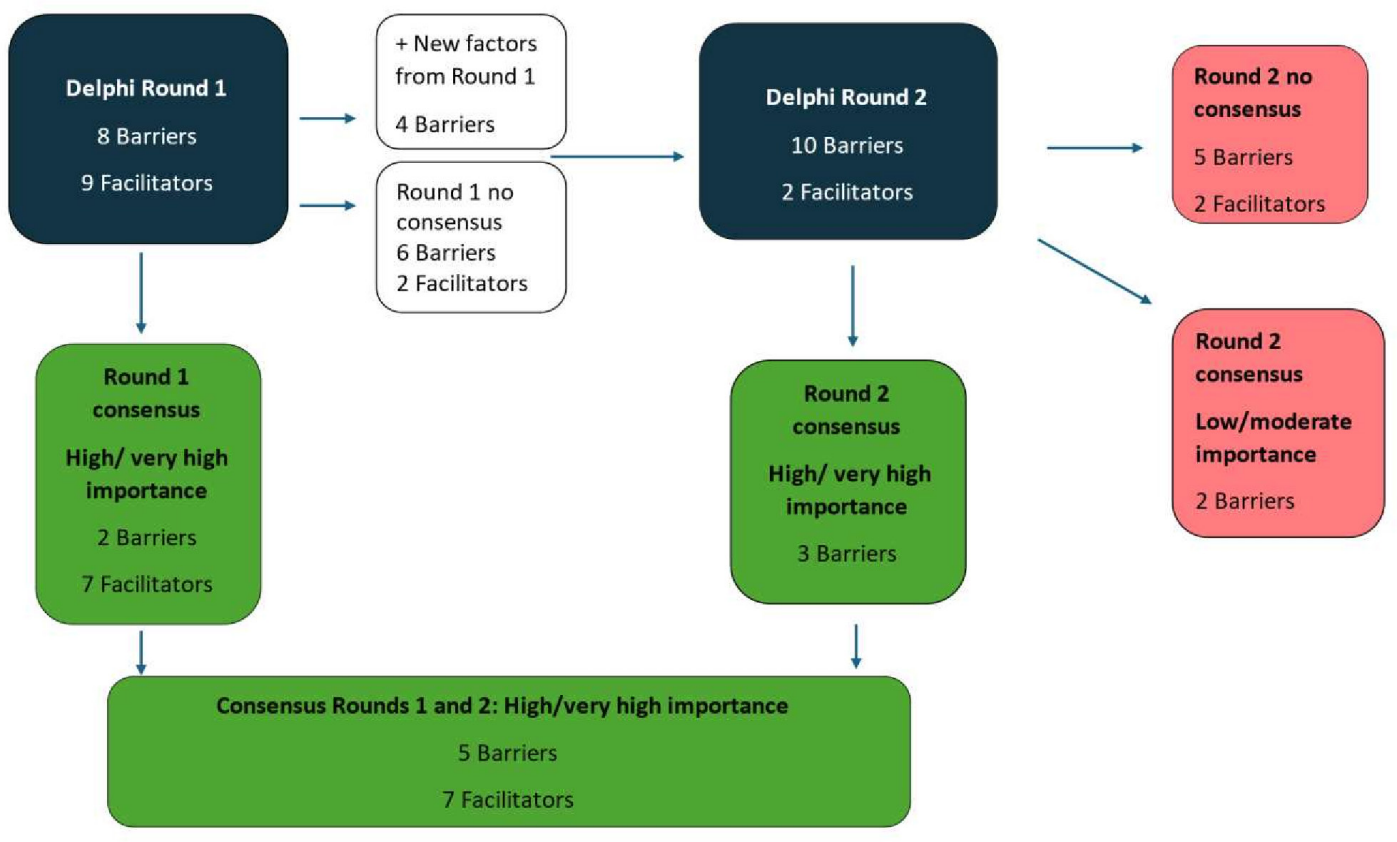
Flow chart of results of two rounds of Delphi survey.

A flow chart of the Delphi rounds can be seen in figure 2. The top five rankings, which were included to provide a method to prioritise the most important factors, should respondents rate all or many factors as important or very important, were not required for prioritisation and are presented in online supplemental file 5.

#### Delphi round 1

The list of 17 factors identified from our previous research included eight barriers and nine facilitators to referral from healthcare workers’ perspectives. Two barriers and seven facilitators reached consensus as being of high/very high importance in round 1 and four new barriers were suggested. One new barrier related to healthcare professionals’ concern about the label of pre-diabetes, and three barriers related specifically to the Irish health service—the restricted access to electronic referrals, concern about limited availability and accessibility of the programme and concerns about the challenge of recruiting staff to run the programme.

#### Delphi round 2

Round 2 consisted of eight of the original factors (six barriers and two facilitators) and the four new barriers. Of the four new barriers suggested in round 1, three reached consensus as being of high/very high importance and one as being of moderate/low importance. One factor from the original list achieved consensus in round 2 as being of moderate/low importance to address to improve referral.

Results from the two rounds can be seen in online supplemental files 6 and 7. From the two rounds of 21 factors (17 factors from round 1 and 4 new factors added in round 2), consensus was achieved on 12 factors (barriers and facilitators) being of high/very high importance to address in a strategy to improve NDPP referral.

The factors considered important for referral operated at multiple levels of the health system: the health service, NDPP providers (NDPP team that develops, administers and delivers the DPP—in Ireland this is a team within the health service), DPP educators and referrers.^5 14^

### Mapping to intervention functions (BCW stage 2)

The 12 factors that reached consensus for importance were mapped to the COM-B (table 2). All three categories (Capability, Opportunity and Motivation) were identified in the mapping with 7 of the 12 factors relating to *physical opportunity* (TDF domain, Environmental context and resources).

**Table 2:** Consensus factors and final BCTs

Factors that achieved consensus as high/very high importance	Barrier/facilitator	Most appropriate BCTs selected by research team	Example
Limited practical information about the availability of the programme (location, dates, times, referral method) among GPs and practice nurses	Barrier	5.3 Information about social and environmental consequences	5.3 GPs and nurses provided with information of consequences of referring (eg, DPP educator informs referrers what happens once they refer patients including detail about the location, times and dates)
Having a healthcare professional, DPP educator or other referrer, promoting the DPP to potential referrers to encourage referral	Facilitator	9.1 Credible source 5.1 Information about health consequences	9.1 DPP educators promote the programme to the referrers 5.1 DPP educators provide information to referrers about the effectiveness of DPPs
Before discussing referral to the DPP, GPs and practice nurses discuss patients’ understanding of pre-diabetes and the value of diabetes prevention	Facilitator	5.3 Information about social and environmental consequences	5.3 DPP educators inform referrers that attenders of the pilot programme viewed the programme as a necessary support for behaviour change and not a burden 5.3 DPP educators inform referrers that patients are more likely to accept referral if recommended by a healthcare professional
Alerts within electronic health records in general practice to prompt screening for pre-diabetes and referral to the DPP	Facilitator	7.1 Prompts/cues	7.1 Health service assists general practice to modify the electronic health record to prompt referrers to screen and refer to the DPP
A clear and easy referral pathway from general practice to the DPP	Facilitator	6.1 Demonstration of the behaviour 4.1 Instruction on how to perform a behaviour 3.2 Social support (practical)	6.1 DPP providers provide information for referrers on how to fill out the referral form/send electronic referrals (eg, a short instructional video) 4.1 DPP educator provides instruction on how to refer 3.2 DPP providers provide practical support to referrers (eg, Zoom call/email/phone call) to address any problems with referral
Electronic referrals from general practice to the DPP to facilitate referral	Facilitator	12.1 Restructuring the physical environment 2.7 Feedback on outcome of behaviour 3.2 Social support (practical)	12.1 Health service continues to enable electronic referrals from referrers to the DPP on the HealthLink system (secure messaging system between primary care and the health service) 2.7 DPP providers notify the referrer that the electronic referral has been received 3.2 DPP providers provide practical support to referrers (eg, zoom call/email/phone call to address any problems with referral)
Where available, electronic referrals are currently restricted to GPs as practice nurses do not have access to Healthlink (secure messaging service between primary care and the health service)	Barrier	12.1 Restructuring the physical environment	12.1 Health service enables practice nurses to refer through Healthlink
Provide referrers with the opportunity to feedback to the DPP providers on the referral process	Facilitator	3.2 Social support (practical)	3.2 Referrers provided with contact details of DPP providers or technical support to provide feedback on any issues encountered
Feedback to the referrer from the DPP providers about their patients after referral, for example, if their patient took up a place on the programme	Facilitator	2.7 Feedback on the outcome of a behaviour	2.7 DPP providers notify the referrer that the referral has been received 2.7 DPP providers send information to the referrer when the patient starts the programme/progress report/discharge report with outcomes
Concerns about recruitment of dietitians to deliver the programme could lead to decreased referrals due to the expectation of long wait time or decreased availability	Barrier	2.7 Feedback on outcome of behaviour 5.3 Information about social and environmental consequences	2.7 DPP providers notify referrers that referral has been received 5.3 If service level staffing issues are causing delays in referral, then DPP providers feedback to referrers on status of referrals, length of waiting list
Long waiting time between referral and enrolment into the DPP	Barrier	2.7 Feedback on outcome of behaviour	2.7 DPP providers notify referrers that referral has been received with feedback to referrers on status of referrals, length of waiting list
Concern about the accessibility and availability of the programme in rural and local areas	Barrier	5.3 Information about social and environmental consequences	5.3 Information on social and environmental consequences of referring (eg, DPP educator informs referrers what happens once they refer including detail about the location, times and dates with programme availability, online option)

BCT, behaviour change technique; DPP, diabetes prevention programme; GP, general practitioner.

For each COM-B category, the corresponding intervention function was selected, giving a list of seven potentially relevant intervention functions (Education, Training, Restriction, Environmental restructuring, Enablement, Persuasion and Modelling). The intervention function *restriction* was removed after the research team discussion, as it was deemed not relevant to the context of referral to the NDPP. Intervention functions were also mapped to potentially useful *policy categories* in the BCW for support at the health system level with all seven categories identified as potentially useful (online supplemental file 8).

### Selecting BCTs (BCW stage 3)

BCTs associated with each of the remaining six intervention functions were identified using guidance in the BCW.^16^ This generated a list of 103 BCTs across the six intervention functions. This long list was reduced to a short list of the most frequently used BCTs.^16 22^ The short list contained a total of 31 BCTs across the six intervention functions, representing 20 unique BCTs (online supplemental file 9). The intervention functions and the shortlist of BCTs were discussed by the research team and the most appropriate intervention functions and BCTs were selected to address each factor within the context of primary healthcare delivery in Ireland, resulting in a final list of nine BCTs reflecting five intervention functions (table 2).

### Proposed strategy

The final strategy to change healthcare professionals’ referral behaviour involves different stakeholders: the referrers (GPs and practice nurses), the DPP providers (DPP team that develops, administers and delivers the DPP—In Ireland, this is a team within the health service), DPP educators and the health service (table 2).

Using the identified BCTs (table 2), the strategy should include education and training delivered by educators (credible source) to the referrers with additional materials that the referrers can access themselves, for example, online material. This education and material should include practical information on the availability of the programme (information about social and environmental consequences) and instruction in how to refer (instruction on how to perform a behaviour) and include evidence of the effectiveness of DPPs (information about health consequences). Two-way communication is encouraged to provide the referrer with information/updates on their patients (feedback on outcome of behaviour, information about social and environmental consequences) and with technical assistance if any questions or issues arise with the referral process (social support (practical)). Practical information on availability and information on waiting times should also be included in the educational material to address referrer concerns about programme availability (information about social and environmental consequences).

The strategy should include modifications to electronic health records to include prompts to the referrer to screen/refer patients to the NDPP (prompts/cues) and electronic referrals should continue to be enabled and expanded for GPs. GPs who sign up to the health service’s Structured Chronic Disease Management Programme have access to electronic referrals; however, the DPP is not yet available in all areas. Consideration should be given by the health service to electronic referral from practice nurses if clinical governance allows.

## Discussion

This study describes the process of prioritising factors affecting referral and identifying content for a strategy to improve referral to an NDPP, using a Delphi survey with healthcare professionals and the BCW approach. The BCTs identified and the proposed strategy are informed by evidence on the barriers and enablers to referral and the strategy is tailored to the Irish healthcare context.^5 14^ By providing a worked example of the stages involved in the development of an implementation strategy, we have added to the limited body of research using theoretically informed processes to improve referral to DPPs. The prioritisation process using the Delphi survey will be a useful guide for researchers seeking to involve stakeholders in the strategy development process.

While the evidence supports the effectiveness of DPPs, referrals to the programmes from healthcare professionals have been suboptimal.^6^ As a result, some research has begun to focus on the development of strategies to improve referrals to DPPs.^11 12 25^ The studies describe strategies such as modifications to the electronic health record to identify and refer people with pre-diabetes, education of potential referrers and a healthcare professional champion, which align with our suggested strategy. An overview of systematic reviews by Chauhan *et al*, evaluating behaviour change interventions and healthcare professionals’ practice in chronic disease management, found that education, training with audit and feedback and enablement through clinical decision support systems and electronic health records were effective to change behaviour^26^ which broadly aligns with our recommendations.

The two factors that reached 100% consensus as being of high/very high importance in our Delphi survey were enabling electronic referrals and a clear and easy referral process. In an implementation study using education, a pre-diabetes champion and the electronic health record to increase referrals to a DPP, clinicians reported that electronic referral made it more likely that they would refer.^11^ A scoping review examining the effectiveness and cost-effectiveness of electronic referrals in healthcare found that electronic referrals had the potential to increase the quality and quantity of referrals and physicians viewed electronic referrals favourably.^27^ Our study highlights that the electronic referral process (as part of the referral pathway) will need to be clear and easy to use. The BCTs identified in our study, namely, feedback on the referral at the individual level and support from the diabetes prevention providers if issues arise, will ensure greater clarity.

The promotion of the programme to referrers by another healthcare professional was prioritised in our Delphi survey. The potential for healthcare professionals to positively influence other healthcare professionals’ behaviour was recognised in a Cochrane review of 69 studies investigating the effects of educational outreach visits (in-person visits by a trained person to change practice within the practice setting).^28^ Our systematic review on healthcare workers’ perspectives on referral indicated that in-person promotion and education by a healthcare professional was valued by potential referrers.^5^ Another Cochrane systematic review of 24 studies assessed the effectiveness of opinion leaders (influential healthcare colleagues) on healthcare professional behaviour and found that opinion leaders can be effective in promoting evidence-based practice.^29^ Promotion of the programme by DPP educators should be considered an integral part of service delivery.

One factor which did not achieve consensus was *alternative referral pathways to the DPP, for example, through community organisations engaging with groups at higher risk of developing type 2 diabetes*. A systematic review to synthesise the research on cultural adaptations and implementation research in DPPs identified 15 studies using numerous cultural adaptations to increase participation of ethnic minority populations.^30^ Community health workers, as trusted community members, were able to bridge the gap between the community and the DPP and a Community Based Participatory Research approach, working within the communities, appeared to facilitate acceptance of the programme. As the NDPP is a new programme in Ireland which is just establishing referral pathways from primary care, future research should investigate the characteristics of attenders and non-attenders to assess if those most at risk are being reached and if alternative pathways are required.

Involving stakeholders in health research has been recommended to increase the uptake of research into practice.^31^ While the BCW provides a useful structure to guide the development of strategies, there is no standard way to involve stakeholders in the process, with studies describing different methods such as expert workshops or Delphi surveys.^23 32^ Involving stakeholders in the identification of BCTs can be challenging due to the academic language used.^24^ In our Delphi survey, we asked healthcare professionals to prioritise the barriers and facilitators to referral and the research team chose the most appropriate BCTs to decrease the respondent burden. Transparently reporting the steps taken to identify BCTs will allow for replication and may be useful for the development of other interventions using the BCW. Next steps in this study would be further consultation with stakeholders to discuss the acceptability, feasibility and operationalisation of the BCTs in the Irish setting.

### Strengths and limitations

This study has a number of strengths. First, using the BCW is a systematic and structured method that encourages the consideration of a wide range of options to change behaviour that are likely to be effective.^16^ The study is aligned with the development phase of the UK Medical Research Council’s updated guidance on complex intervention development, which includes involving stakeholders, having a development team with expertise, reviewing existing literature, including primary data, and consideration of the context.^31^ Using the Delphi survey to prioritise the barriers and facilitators was another strength. Delphi surveys have been widely used in healthcare to achieve consensus and develop evidence-based recommendations, and the anonymity provided by the Delphi survey decreases the risk of dominance by one group or individual.^33^

The original list of factors affecting referral, that were prioritised in the Delphi, was drawn from a systematic review of 37 studies conducted in the USA, England and Australia^5^ and a qualitative study from Ireland^14^ which are all higher-income countries. These findings could be applied to private (USA), public (UK) or mixed public and private healthcare systems (Ireland and Australia). The importance of the referral pathway (clear and easy pathway, electronic referral), healthcare professional workload and resources (time and staff) and concern about the burden of attending (length of time involved) were important factors across all systems. Concern about the financial cost of the programme and insurance cover was an important factor in the USA.^5^ The factors from the original list prioritised by Irish healthcare professionals in the Delphi survey are specific to the Irish primary care context at the time of the survey; however, they may be applicable to other healthcare systems. In Ireland, the majority of the population pays to access services in primary care, including GPs who are gatekeepers for a range of primary and secondary care services.^34^ An ageing population and the development of new technologies and treatments are increasing the demand for services, and this, coupled with a healthcare workforce shortage, can result in long waiting times to access care.^34^ Reforms in the Irish healthcare service are aiming to increase care in the community with chronic disease management and prevention programmes accessed through GPs, including the NDPP. While contextual to Ireland, the factors prioritised in the Delphi survey could therefore be generalisable to health systems rolling out DPPs that have healthcare workforce shortages, mixed public and private healthcare systems and systems experiencing increasing demand for services.

The BCW necessitates that the researchers use their own judgement at various times during the process.^16^ While this was a challenge, we have detailed the decisions made throughout the process to increase transparency and involved a wide range of stakeholders in developing and prioritising the factors important to the Irish context. Another limitation was the attrition during the survey, with a 62% response rate to round 2. To reduce bias, we recruited our panel through healthcare professional organisations rather than the non-random method frequently used in Delphi surveys which is to directly contact the expert panel and invite them to the study.^20^ This may have affected the response rate and attrition between rounds is a limitation of Delphi studies.^20^ There are no recommended guidelines for an acceptable rate of attrition; however, there are recommendations on ways to minimise it.^20^ Every effort was made to increase response rates in our study with the respondents involved in the selection of barriers and facilitators rather than BCTs, limiting the response time to 10 min, a short time between rounds, limiting the number of rounds and sending reminders.

One final limitation is that data on the practice settings of the Delphi respondents were not collected. The characteristics of the practice (eg, rural vs urban setting, practice size, characteristics of the communities) may have influenced perspectives on the barriers and facilitators faced. A 2025 systematic review of healthcare workers’ perceptions on referral to DPPs found referral more difficult in more rural, less populated areas or areas with poor transportation.^5^ A qualitative study on clinicians’ perspectives of diabetes prevention reported that the high prevalence of diabetes in a resource-constrained safety net healthcare setting created an expectation to prioritise the treatment of diabetes over prevention.^35^ Future research could explore the influence of contextual factors on healthcare professionals’ perspectives to identify modifiable factors to improve referral to DPPs in different healthcare settings.

## Conclusion

This study is one of the few to use a theoretically informed approach to develop a strategy to improve referral to an NDPP. We prioritised factors affecting referral and systematically identified BCTs to change health professional behaviour, an under-researched area. Nine BCTs were identified to include in a strategy to improve referrals. This study will be useful for researchers, healthcare professionals and health systems seeking to improve referral to DPPs and to researchers seeking transparency on the intervention development process.

## Supplementary material

10.1136/bmjopen-2025-104300 online supplemental file 1

## Data Availability

All data relevant to the study are included in the article or uploaded as supplementary information.
