# Supplementary material for: Designing an implementation strategy to improve referral from general practice to a National Diabetes Prevention Programme using a Delphi survey with healthcare professionals and the Behaviour Change Wheel
Source: BMJ Open. 2026 Feb 16;16(2):e104300. doi: 10.1136/bmjopen-2025-104300 (PMC12911679; doi:10.1136/bmjopen-2025-104300)
Supplement: online supplemental file 1 [file bmjopen-16-2-s001.docx]

**Supplementary File 1**_Table of survey questions, origin of question and TDF Domains

|  |  | **Supplementary File 1: Table of survey questions, origin of question and TDF Domains** | | | |
| --- | --- | --- | --- | --- | --- |
| Item | Survey Question Number | **How would you rate the importance of each factor in *hindering* the referral to the national DPP** | Findings from systematic review | Findings from qualitative study | TDF Domains |
| 1 | 7 | Limited information on the effectiveness of DPPs among GPs and practice nurses | x |  | Knowledge |
| 2 | 8 | Limited practical information about the availability of the programme (location, dates, times, referral method) among GPs and practice nurses | x |  | Knowledge |
| 3 | 9 | GPs’ and practice nurses’ concern about the additional workload involved in identifying and referring people to the DPP | x |  | Environmental context and resources |
| 4 | 10 | A long waiting time between referral and enrolment into the DPP | x |  | Environmental context and resources |
| 5 | 11 | GPs and practice nurses concerns that their patients will not attend due to the time commitment involved | x |  | Expectation of patient barriers |
| 6 | 12 | Concerns by referrers and educators that patients will not attend because of the lag between the diagnosis of prediabetes and referral into the DPP | x |  | Expectation of patient barriers |
| 7 | 13 | Referral to the DPP not prioritised when there are competing medical needs | x |  | Environmental context and resources |
| 8 | 14 | GPs’ and practice nurses’ perceptions about their patient’s low motivation to attend the diabetes prevention programme | x |  | Expectation of patient barriers |
|  |  | **How would you rate the importance of each factor in *helping* the referral process** |  |  |  |
| 9 | 15 | Having a healthcare professional, DPP educator or other referrer, promoting the DPP to potential referrers | x | x | Social professional role and identity |
| 10 | 16 | Financial incentives for general practice to support referral to the DPP | x |  | Reinforcement |
| 11 | 17 | A clear and easy referral pathway from general practice to the DPP | x | x | Environmental context and resources |
| 12 | 18 | Alerts within electronic health records in general practice to prompt screening for prediabetes and referral to the DPP | x |  | Environmental context and resources |
| 13 | 19 | Electronic referrals from general practice to the DPP to facilitate referral | x | x | Environmental context and resources |
| 14 | 20 | Feedback to the referrer from the DPP providers about their patients after referral, for example, if their patient took up a place on the programme | x |  | Environmental context and resources |
| 15 | 21 | Opportunities for referrers to feedback to the DPP providers on the referral process | x |  | Environmental context and resources |
| 16 | 22 | Alternative referral pathways to the DPP, for example, through community organisations engaging with groups at higher risk of developing type 2 diabetes | x |  | Environmental context and resources |
| 17 | 23 | Before discussing referral to the DPP, GPs and practice nurses discuss patients’ understanding of prediabetes and the value of diabetes prevention | x | x | “Other” /Expectation of patient barriers |

DPP- Diabetes prevention programme, TDF – Theoretical Domains Framework, GP - General Practitioner

**Supplementary File 2:** Summary of barriers and recommendations to referral to DPPs from HCW’s perspectives (Haseldine et al, 2025)

| Barrier | Recommendations |
| --- | --- |
| Referral pathway unclear to potential referrers | - DPP providers inform potential referrers of the criteria for referral and how to refer - DPP providers use existing pathways (eg primary care) for referral - DPP facilitates electronic referrals from referrers - DPP providers use a standard easy referral process - DPP providers pilot referral pathway with potential referrers |
| Ethical issues using electronic health record to refer people who were unaware of their prediabetes status | - Involve healthcare professionals in the referral process |
| Difficulty engaging those at high risk who do not use primary care | - Consider alternate pathways such as pharmacies or community groups - Clarify roles within the referral process |
| Potential referrers lack the time or staff to identify and refer people to DPPs | - Prediabetes champion - Additional clinical staff - Financial incentives for referrers - Administrative support for referral |
| Lack of availability of DPPs or long waiting times after referral | - Increasing availability of DPPs - Virtual DPPs - Provide information to referrers on their referrals and waiting times |
| Uncertainty about insurance cover | - Provide insurance cover for DPPs - Simplify process of applying for insurance cover - Inform referrers about the insurance cover available |
| Referrers perception of time and financial burden on their patients | - Inform referrers about available insurance cover for DPPs (e.g. Medicaid and Medicare) - Reframe the DPP as support for behaviour change |
| Low awareness of prediabetes and diabetes prevention among people with prediabetes | - Referrers discuss risk and the effectiveness of DPPs before referral |
| Lack of knowledge of DPPs among potential referrers | - Personal contact from DPP provider or other healthcare professional to promote the programme and to inform potential referrers about the effectiveness of the programme |

DPPs - Diabetes Prevention Programmes, HCWs – Healthcare Workers

Reference: Haseldine C, Pallin JA, Kearney PM, Mc Hugh SM, Riordan F, Cotterill S, O'Donoghue G. Healthcare workers' perspectives on barriers and facilitators to referral to type 2 diabetes prevention programmes: a systematic review. BMJ Open. 2025 Mar 7;15(3):e090105. doi: 10.1136/bmjopen-2024-090105.

**Supplementary File 3:** The Theoretical Domains Framework mapped to the sub-constructs of capability, opportunity and motivation from the Capability, Opportunity, Motivation – Behaviour (COM-B) model.


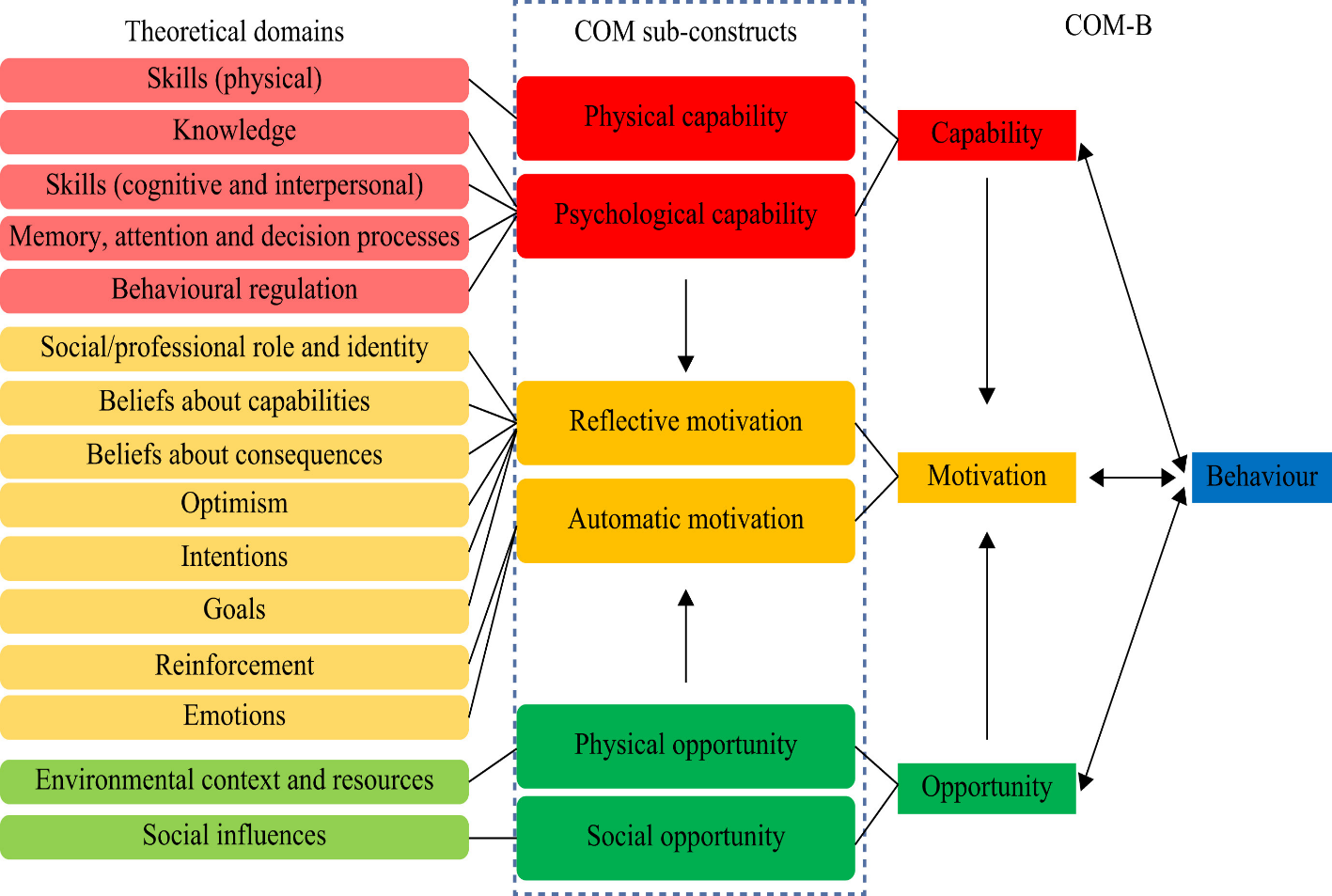


From: Chater AM, Family H, Abraao LM, Burnett E, Castro-Sanchez E, Du Toit B, Gallagher R, Gotterson F, Manias E, Mcewen J, de Figueiredo RM. Influences on nurses' engagement in antimicrobial stewardship behaviours: a multi-country survey using the Theoretical Domains Framework. Journal of Hospital Infection. 2022 Nov 1;129:171-80.

**Supplementary File 4 COREQ checklist**

**
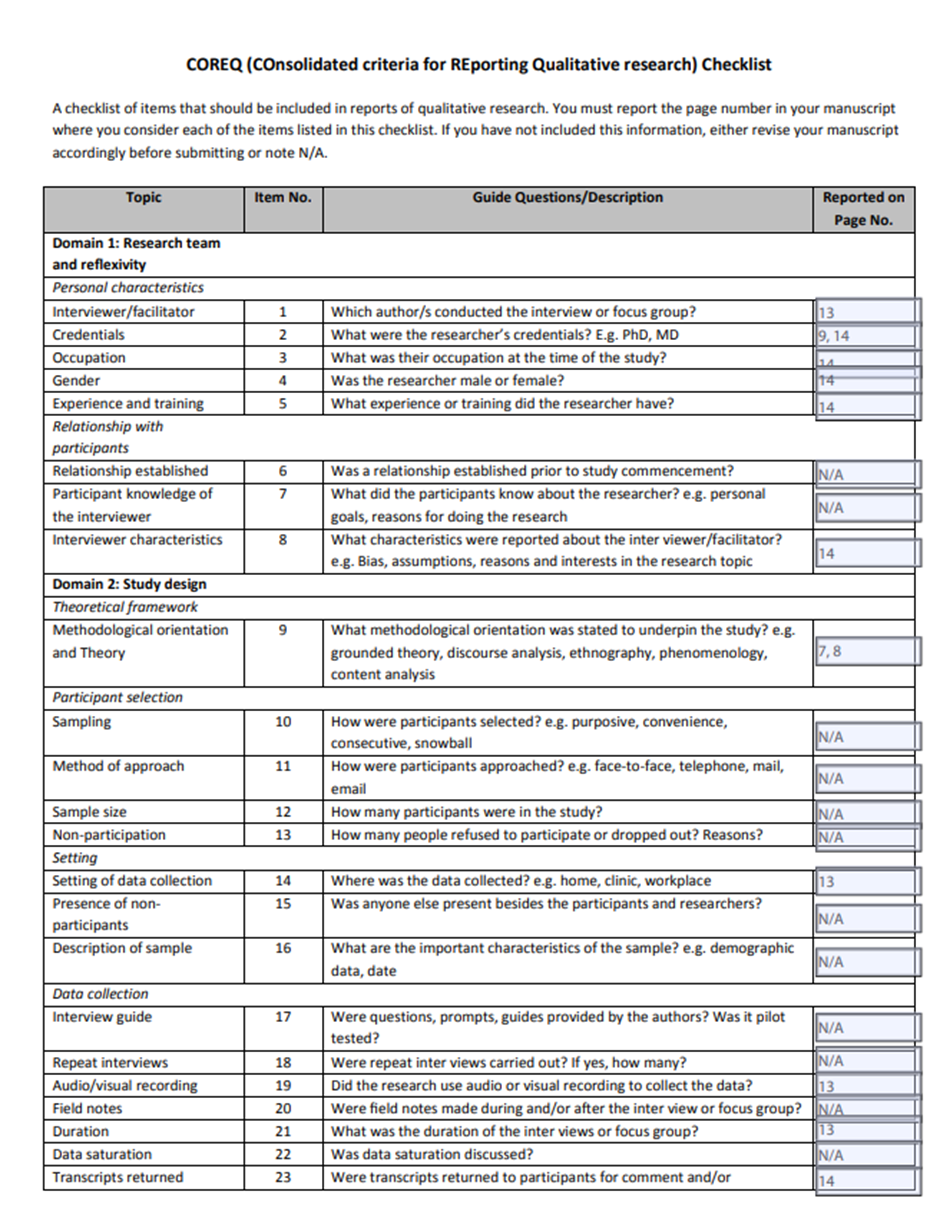
**


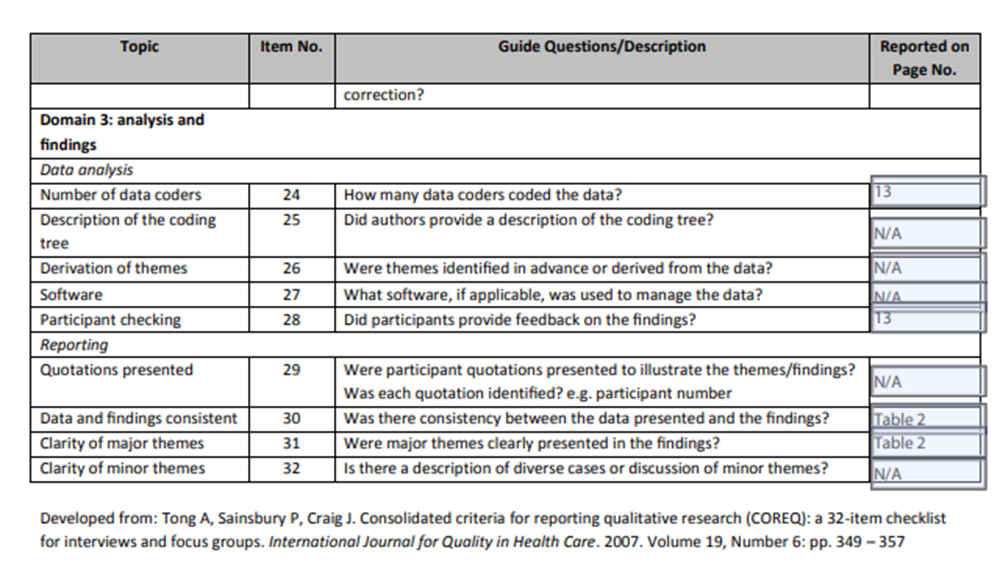


Supplementary File 5_Table of Top 5 Round 1 and Round 2

| **Supplementary File 2: Table of Top 5 Round 1 and Round 2** | | | | |
| --- | --- | --- | --- | --- |
|  | **Round 1** | | **Round 2** | |
| **Factors affecting referral** | **% ranked in top 5** | **Overall rank (Weighted ranking)** | **% ranked in top 5** | **Overall rank**  **(Weighted ranking)** |
| Limited information on the effectiveness of DPPs provided to GPs and practice nurses | 10.8 | 14 (12) | 38.4 | 7 (27) |
| Limited practical information about the availability of the programme (location, dates, times, referral method) among GPs and practice nurses | 56.8 | 2 (71) |  |  |
| GPs’ and practice nurses’ concern about the additional workload involved in identifying and referring people to the DPP | 32.4 | 4 (40) | 56.5 | 3 (42) |
| Long waiting time between referral and enrolment into the DPP | 37.8 | 5 (39) |  |  |
| GPs’ and practice nurses’ concerns that their patients will not attend the DPP due to the time commitment involved | 16.2 | 11 (15) | 39.1 | 7 (27) |
| Concerns by referrers and educators that patients will not attend because of the lag between the diagnosis of prediabetes and referral into the DPP | 16.2 | 12 (14) | 52.2 | 4 (34) |
| Referral to the DPP not prioritised when there are competing medical needs | 18.9 | 9 (21) | 60.9 | 5 (28) |
| GPs’ and practice nurses’ perceptions about their patient’s low motivation to attend the DPP | 10.8 | 15 (11) | 17.4 | 11 (7) |
| Having a healthcare professional, DPP educator or other referrer, promoting the DPP to potential referrers | 29.7 | 8 (26) |  |  |
| Financial incentives for general practice to support referral to the DPP | 18.9 | 10 (20) | 30.4 | 9 (13) |
| A clear and easy referral pathway from general practice to the DPP | 83.8 | 1 (117) |  |  |
| Alerts within electronic health records in general practice to prompt screening for prediabetes and referral to the DPP | 27 | 7 (27) |  |  |
| Electronic referrals from general practice to the DPP to facilitate referral | 51.4 | 3 (67) |  |  |
| Feedback to the referrer from the DPP providers about their patients after referral, for example, if their patient took up a place on the programme | 21.6 | 13 (13) |  |  |
| Opportunities for referrers to feedback to the DPP providers on the referral process | 8.1 | 16 (4) |  |  |
| Alternative referral pathways to the DPP, for example, through community organisations engaging with groups at higher risk of developing type 2 diabetes | 16.2 | 12 (14) | 26.1 | 8 (16) |
| Before discussing referral to the DPP, GPs and practice nurses discuss patients’ understanding of prediabetes and the value of diabetes prevention | 29.7 | 6 (29) |  |  |
| Healthcare professionals concern about the label of prediabetes |  |  | 8.7 | 10 (8) |
| Where available electronic referrals restricted to GPs as practice nurses do not have access to Healthlink (secure messaging system between primary care and the health service) |  |  | 65.2 | 1 (59) |
| Concerns about recruitment of dietitians to deliver the programme leading to decreased referrals due to expectation of long wait time or decreased availability |  |  | 56.5 | 2 (45) |
| Concern about the accessibility and availability of the programme in rural and local areas |  |  | 39.1 | 6 (29) |

DPP- Diabetes prevention programme, GP General Practitioner

Supplementary File 6: Results Delphi Round 1 on the importance of factors hindering referral to the National Diabetes Prevention Programme

| **Round 1 (n=37)** | | | | |
| --- | --- | --- | --- | --- |
| **Importance of each factor in *hindering* referral to NDPP** | **Not important** | **Low/Moderate importance**  **%** | **High/ Very high importance**  **%** | **Missing**  **%** |
| Limited information on the effectiveness of DPPs provided to GPs and practice nurses | 0 | 40.5 | 59.4 |  |
| Limited practical information about the availability of the programme (location, dates, times, referral method) among GPs and practice nurses | 0 | 16.2 | 83.7 |  |
| GPs’ and practice nurses’ concern about the additional workload involved in identifying and referring people to the DPP | 0 | 56.7 | 43.2 |  |
| A long waiting time between referral and enrolment into the DPP | 0 | 21.6 | 78.4 |  |
| GPs’ and practice nurses’ concerns that their patients will not attend the DPP due to the time commitment involved | 0 | 51.3 | 48.6 |  |
| Concerns by referrers and educators that patients will not attend because of the lag between the diagnosis of prediabetes and referral into the DPP | 2.7 | 48.6 | 48.6 |  |
| Referral to the DPP not prioritised when there are competing medical needs | 8.1 | 45.9 | 43.2 | 2.7 |
| GPs’ and practice nurses’ perceptions about their patient’s low motivation to attend the DPP | 0 | 54 | 43.2 | 2.7 |
| **Importance of each factor in *helping* referral to the NDPP** | **Not important** | **Low/Moderate importance**  **%** | **High/ Very high importance**  **%** | **Missing**  **%** |
| Having a healthcare professional, DPP educator or other referrer, promoting the DPP to potential referrers | 0 | 10.8 | 89.1 |  |
| Financial incentives for general practice to support referral to the DPP | 5.4 | 51.3 | 40.5 | 2.7 |
| A clear and easy referral pathway from general practice to the DPP | 0 | 0 | 100 |  |
| Alerts within electronic health records in general practice to prompt screening for prediabetes and referral to the DPP | 0 | 13.5 | 86.5 |  |
| Electronic referrals from general practice to the DPP to facilitate referral | 0 | 0 | 100 |  |
| Feedback to the referrer from the DPP providers about their patients after referral, for example, if their patient took up a place on the programme | 0 | 10.8 | 89.1 |  |
| Provide referrers with opportunity to feedback to the DPP providers on the referral process | 0 | 27 | 72.9 |  |
| Alternative referral pathways to the DPP, for example, through community organisations engaging with groups at higher risk of developing type 2 diabetes | 0 | 43.2 | 56.7 |  |
| Before discussing referral to the DPP, GPs and practice nurses discuss patients’ understanding of prediabetes and the value of diabetes prevention | 0 | 16.2 | 83.7 |  |

DPP – diabetes prevention programme, GP – General Practitioner

Supplementary File 7: Results Delphi Round 2 on the importance of factors hindering referral to the DPP

| **Round 2 (n=23)** | | | |
| --- | --- | --- | --- |
| **Importance of each factor in *hindering* referral to the National DPP** | **Not important** | **Low/Moderate importance**  **%** | **High/ Very high importance**  **%** |
| Limited information on the effectiveness of DPPs among GPs and practice nurses | 0 | 34.7 | 65.2 |
| GPs’ and practice nurses’ concern about the additional workload involved in identifying and referring people to the DPP | 0 | 56.5 | 43.5 |
| GPs’ and practice nurses’ concerns that their patients will not attend the DPP due to the time commitment involved | 0 | 52.2 | 47.8 |
| Concerns by referrers and educators that patients will not attend because of the lag between the diagnosis of prediabetes and referral into the DPP | 0 | 52.1 | 47.8 |
| Referral to the DPP not prioritised when there are competing medical needs | 0 | 56.5 | 43.5 |
| GPs’ and practice nurses’ perceptions about their patient’s low motivation to attend the DPP | 0 | 73.9 | 26 |
| Healthcare professionals concern about the label of prediabetes | 8.7 | 78.3 | 13 |
| Where available, electronic referrals restricted to GPs as practice nurses do not have access to Healthlink (secure messaging service between primary care and the health service) | 0 | 21.7 | 78.2 |
| Concerns about recruitment of dietitians to deliver the programme could lead to decreased referrals due to expectation of long wait time or decreased availability | 0 | 8.7 | 91.3 |
| Concern about the accessibility and availability of the programme in rural and local areas | 0 | 21.7 | 78.3 |
| **Importance of each factor in *helping* referral to the national DPP** | **Not important**  **%** | **Low/Moderate importance**  **%** | **High/ Very high importance**  **%** |
| Financial incentives for general practice to support referral to the DPP | 8.7 | 65.2 | 26 |
| Alternative referral pathways to the DPP, for example, through community organisations engaging with groups at higher risk of developing type 2 diabetes | 0 | 47.8 | 52.2 |

DPP – Diabetes Prevention Programme, GP – General Practitioner

Supplementary File 8_Table of factors linked to intervention functions and policy categories

| **No** | **Factors that achieved consensus as high/very high importance** | **Intervention Functions** | **Linked to potentially useful policy categories** |
| --- | --- | --- | --- |
| **1** | Limited practical information about the availability of the programme (location, dates, times, referral method) among GPs and practice nurses | **Education**-  educate about ways of enacting the desired behaviour | **Communication/marketing**  **Guidelines**  **Regulation**  **Legislation**  **Service provision** |
| **2** | A clear and easy referral pathway from general practice to the DPP | **Training** – imparting skills  **Environmental restructuring** – changing the physical or social context  **Enablement –** increasing means/ reducing barriers to increase capability (beyond education and training) or opportunity (beyond environmental restructuring) | **Guidelines**  **Fiscal measures**  **Regulation**  **Legislation**  **Service provision**  **Guidelines**  **Fiscal measures**  **Regulation**  **Legislation**  **Environmental/ social planning**  **Guidelines**  **Fiscal measures**  **Regulation**  **Legislation**  **Environmental/ social planning**  **Service provision** |
| **3** | Alerts within electronic health records in general practice to prompt screening for prediabetes and referral to the DPP | **Training** – imparting skills  **Environmental restructuring** – changing the physical or social context  **Enablement –** increasing means/ reducing barriers to increase capability (beyond education and training) or opportunity (beyond environmental restructuring) | **Guidelines**  **Fiscal measures**  **Regulation**  **Legislation**  **Service provision**  **Guidelines**  **Fiscal measures**  **Regulation**  **Legislation**  **Environmental/ social planning**  **Guidelines**  **Fiscal measures**  **Regulation**  **Legislation**  **Environmental/ social planning**  **Service provision** |
| **4** | Electronic referrals from general practice to the DPP | **Training** – imparting skills  **Environmental restructuring** – changing the physical or social context  **Enablement –** increasing means/ reducing barriers to increase capability (beyond education and training) or opportunity (beyond environmental restructuring) | **Guidelines**  **Fiscal measures**  **Regulation**  **Legislation**  **Service provision**  **Guidelines**  **Fiscal measures**  **Regulation**  **Legislation**  **Environmental/ social planning**  **Guidelines**  **Fiscal measures**  **Regulation**  **Legislation**  **Environmental/ social planning**  **Service provision** |
| **5** | Where available electronic referrals restricted to GPs as practice nurses do not have access to electronic referral system | **Training** – imparting skills  **Environmental restructuring** – changing the physical or social context  **Enablement –** increasing means/ reducing barriers to increase capability (beyond education and training) or opportunity (beyond environmental restructuring) | **Guidelines**  **Fiscal measures**  **Regulation**  **Legislation**  **Service provision**  **Guidelines**  **Fiscal measures**  **Regulation**  **Legislation**  **Environmental/ social planning**  **Guidelines**  **Fiscal measures**  **Regulation**  **Legislation**  **Environmental/ social planning**  **Service provision** |
| **6** | Opportunities for referrers to feedback to the DPP providers on the referral process | **Training** – imparting skills  **Environmental restructuring** – changing the physical or social context  **Enablement –** increasing means/ reducing barriers to increase capability (beyond education and training) or opportunity (beyond environmental restructuring) | **Guidelines**  **Fiscal measures**  **Regulation**  **Legislation**  **Service provision**  **Guidelines**  **Fiscal measures**  **Regulation**  **Legislation**  **Environmental/ social planning**  **Guidelines**  **Fiscal measures**  **Regulation**  **Legislation**  **Environmental/ social planning**  **Service provision** |
| **7** | Concerns about recruitment of dietitians to deliver the programme leading to decreased referrals due to expectation of long wait time or decreased availability | **Education** - educate about ways of enacting the desired behaviour  **Persuasion** – using communication to induce positive or negative feelings or stimulate action  **Modelling** – providing an example for people to aspire to or imitate | **Communication/marketing**  **Guidelines**  **Regulation**  **Legislation**  **Service provision**  **Communication/marketing**  **Guidelines**  **Regulation**  **Legislation**  **Service provision**  **Communication/marketing**  **Service provision** |
| **8** | Concern about the accessibility and availability of the programme in rural and local areas | **Education**-  educate about ways of enacting the desired behaviour | **Communication/marketing**  **Guidelines**  **Regulation**  **Legislation**  **Service provision** |
| **9** | Long waiting time between referral and enrolment into the DPP | **Training** – imparting skills  **Environmental restructuring** – changing the physical or social context  **Enablement –** increasing means/ reducing barriers to increase capability (beyond education and training) or opportunity (beyond environmental restructuring) | **Guidelines**  **Fiscal measures**  **Regulation**  **Legislation**  **Service provision**  **Guidelines**  **Fiscal measures**  **Regulation**  **Legislation**  **Environmental/ social planning**  **Guidelines**  **Fiscal measures**  **Regulation**  **Legislation**  **Environmental/ social planning**  **Service provision** |
| **10** | Feedback to the referrer from the DPP providers about their patients after referral, for example, if their patient took up a place on the programme | **Training** – imparting skills  **Environmental restructuring** – changing the physical or social context  **Enablement –** increasing means/ reducing barriers to increase capability (beyond education and training) or opportunity (beyond environmental restructuring) | **Guidelines**  **Fiscal measures**  **Regulation**  **Legislation**  **Service provision**  **Guidelines**  **Fiscal measures**  **Regulation**  **Legislation**  **Environmental/ social planning**  **Guidelines**  **Fiscal measures**  **Regulation**  **Legislation**  **Environmental/ social planning**  **Service provision** |
| **11** | Before discussing referral to the DPP, GPs and practice nurses discuss patients’ understanding of prediabetes and the value of diabetes prevention | **Education**-  educate about ways of enacting the desired behaviour | **Communication/marketing**  **Guidelines**  **Regulation**  **Legislation**  **Service provision** |
| **12** | Having a healthcare professional, DPP educator or other referrer, promoting the DPP to potential referrers | **Education** - educate about ways of enacting the desired behaviour  **Persuasion** – using communication to induce positive or negative feelings or stimulate action  **Modelling** – providing an example for people to aspire to or imitate | **Communication/marketing**  **Guidelines**  **Regulation**  **Legislation**  **Service provision**  **Communication/marketing**  **Guidelines**  **Regulation**  **Legislation**  **Service provision**  **Communication/marketing**  **Service provision** |

Supplementary File 9_Consensus factors and the BCW

| **Supplementary File 4: Consensus factors and the BCW** | | | | | |
| --- | --- | --- | --- | --- | --- |
| **Factors that achieved consensus as high/very high importance** | **Barrier/**  **Facilitator** | **TDF** | **COM B** | **Intervention Functions** | **BCTs**  **Most frequently used** |
| Limited practical information about the availability of the programme (location, dates, times, referral method) among GPs and practice nurses | Barrier | Knowledge | **Capability**  Psychological | **Education**-  educate about ways of enacting the desired behaviour | 5.3 Information about social and environmental consequences  5.1 Information about health consequences  2.2 Feedback on behaviour  2.7 Feedback on outcome of behaviour  7.1 Prompts/cues  2.3 Self- monitoring of behaviour |
| Having a healthcare professional, DPP educator or other referrer, promoting the DPP to potential referrers to encourage referral | Facilitator | Social professional role and identity | **Motivation**  Reflective | **Education** - educate about ways of enacting the desired behaviour  **Persuasion** – using communication to induce positive or negative feelings or stimulate action  **Modelling** – providing an example for people to aspire to or imitate | 5.3 Information about social and environmental consequences  5.1 Information about health consequences  2.2 Feedback on behaviour  2.7 Feedback on outcome of behaviour  7.1 Prompts/cues  2.3 Self- monitoring of behaviour  9.1 Credible source  5.3 Information about social and environmental consequences  5.1 Information about health consequences  2.2 Feedback on behaviour  2.7 Feedback on outcome(s) of the behaviour  6.1 Demonstration of the behaviour |
| Before discussing referral to the DPP, GPs and practice nurses discuss patients’ understanding of prediabetes and the value of diabetes prevention | Facilitator | “Other” Domain Expectation of patient barriers  (Beliefs about Consequences of referring) | **Motivation**  Reflective | **Education** - educate about ways of enacting the desired behaviour  **Persuasion** – using communication to induce positive or negative feelings or stimulate action  **Modelling** – providing an example for people to aspire to or imitate | 5.3 Information about social and environmental consequences  5.1 Information about health consequences  2.2 Feedback on behaviour  2.7 Feedback on outcome of behaviour  7.1 Prompts/cues  2.3 Self- monitoring of behaviour  9.1 Credible source  5.3 Information about social and environmental consequences  5.1 Information about health consequences  2.2 Feedback on behaviour  2.7 Feedback on outcome(s) of the behaviour  6.1 Demonstration of the behaviour |
| Alerts within electronic health records in general practice to prompt screening for prediabetes and referral to the DPP | Facilitator | Environmental context and resources | **Opportunity**  Physical | **Training** – imparting skills  **Environmental restructuring** – changing the physical or social context  **Enablement –** increasing means/ reducing barriers to increase capability (beyond education and training) or opportunity (beyond environmental restructuring) | 6.1 Demonstration of the behaviour  4.1 Instruction on how to perform a behaviour  2.2 Feedback on behaviour  2.7 Feedback on outcome of behaviour  2.3 Self- monitoring of behaviour  8.1 Behavioural practice/ rehearsal  12.5 Adding objects to the environment  7.1 Prompts/ cues  12.1 Restructuring the physical environment  3.1 Social support (unspecified)  3.2 Social support (practical)  1.1 Goal setting (behaviour)  1.3 Goal setting (outcome)  12.5 Adding objects to the environment  1.2 Problem solving  1.4 Action planning  2.3 Self-monitoring of behaviour  12.1 Restructuring of physical environment  1.5 Review behaviour goal(s)  1.7 Review outcome goal(s) |
| A clear and easy referral pathway from general practice to the DPP | Facilitator | Environmental context and resources | **Opportunity**  Physical | **Training** – imparting skills  **Environmental restructuring** – changing the physical or social context  **Enablement –** increasing means/ reducing barriers to increase capability (beyond education and training) or opportunity (beyond environmental restructuring) | 6.1 Demonstration of the behaviour  4.1 Instruction on how to perform a behaviour  2.2 Feedback on behaviour  2.7 Feedback on outcome of behaviour  2.3 Self- monitoring of behaviour  8.1 Behavioural practice/ rehearsal  12.5 Adding objects to the environment  7.1 Prompts/ cues  12.1 Restructuring the physical environment  3.1 Social support (unspecified)  3.2 Social support (practical)  1.1 Goal setting (behaviour)  1.3 Goal setting (outcome)  12.5 Adding objects to the environment  1.2 Problem solving  1.4 Action planning  2.3 Self-monitoring of behaviour  12.1 Restructuring of physical environment  1.5 Review behaviour goal(s)  1.7 Review outcome goal(s) |
| Electronic referrals from general practice to the DPP to facilitate referral | Facilitator | Environmental context and resources | **Opportunity**  Physical | **Training** – imparting skills  **Environmental restructuring** – changing the physical or social context  **Enablement –** increasing means/ reducing barriers to increase capability (beyond education and training) or opportunity (beyond environmental restructuring) | 6.1 Demonstration of the behaviour  4.1 Instruction on how to perform a behaviour  2.2 Feedback on behaviour  2.7 Feedback on outcome of behaviour  2.3 Self- monitoring of behaviour  8.1 Behavioural practice/ rehearsal  12.5 Adding objects to the environment  7.1 Prompts/ cues  12.1 Restructuring the physical environment  3.1 Social support (unspecified)  3.2 Social support (practical)  1.1 Goal setting (behaviour)  1.3 Goal setting (outcome)  12.5 Adding objects to the environment  1.2 Problem solving  1.4 Action planning  2.3 Self-monitoring of behaviour  12.1 Restructuring of physical environment  1.5 Review behaviour goal(s)  1.7 Review outcome goal(s) |
| Where available electronic referrals currently restricted to GPs as practice nurses do not have access to Healthlink (electronic referral system) | Barrier | Environmental context and resources | **Opportunity**  Physical | **Training** – imparting skills  **Environmental restructuring** – changing the physical or social context  **Enablement –** increasing means/ reducing barriers to increase capability (beyond education and training) or opportunity (beyond environmental restructuring) | 6.1 Demonstration of the behaviour  4.1 Instruction on how to perform a behaviour  2.2 Feedback on behaviour  2.7 Feedback on outcome of behaviour  2.3 Self- monitoring of behaviour  8.1 Behavioural practice/ rehearsal  12.5 Adding objects to the environment  7.1 Prompts/ cues  12.1 Restructuring the physical environment  3.1 Social support (unspecified)  3.2 Social support (practical)  1.1 Goal setting (behaviour)  1.3 Goal setting (outcome)  12.5 Adding objects to the environment  1.2 Problem solving  1.4 Action planning  2.3 Self-monitoring of behaviour  12.1 Restructuring of physical environment  1.5 Review behaviour goal(s)  1.7 Review outcome goal(s) |
| Provide referrers with opportunity to feedback to the DPP providers on the referral process | Facilitator | Environmental context and resources | **Opportunity**  Physical | **Training** – imparting skills  **Environmental restructuring** – changing the physical or social context  **Enablement –** increasing means/ reducing barriers to increase capability (beyond education and training) or opportunity (beyond environmental restructuring) | 6.1 Demonstration of the behaviour  4.1 Instruction on how to perform a behaviour  2.2 Feedback on behaviour  2.7 Feedback on outcome of behaviour  2.3 Self- monitoring of behaviour  8.1 Behavioural practice/ rehearsal  12.5 Adding objects to the environment  7.1 Prompts/ cues  12.1 Restructuring the physical environment  3.1 Social support (unspecified)  3.2 Social support (practical)  1.1 Goal setting (behaviour)  1.3 Goal setting (outcome)  12.5 Adding objects to the environment  1.2 Problem solving  1.4 Action planning  2.3 Self-monitoring of behaviour  12.1 Restructuring of physical environment  1.5 Review behaviour goal(s)  1.7 Review outcome goal(s) |
| Feedback to the referrer from the DPP providers about their patients after referral, for example, if their patient took up a place on the programme | Facilitator | Environmental context and resources | **Opportunity**  Physical | **Training** – imparting skills  **Environmental restructuring** – changing the physical or social context  **Enablement –** increasing means/ reducing barriers to increase capability (beyond education and training) or opportunity (beyond environmental restructuring) | 6.1 Demonstration of the behaviour  4.1 Instruction on how to perform a behaviour  2.2 Feedback on behaviour  2.7 Feedback on outcome of behaviour  2.3 Self- monitoring of behaviour  8.1 Behavioural practice/ rehearsal  12.5 Adding objects to the environment  7.1 Prompts/ cues  12.1 Restructuring the physical environment  3.1 Social support (unspecified)  3.2 Social support (practical)  1.1 Goal setting (behaviour)  1.3 Goal setting (outcome)  12.5 Adding objects to the environment  1.2 Problem solving  1.4 Action planning  2.3 Self-monitoring of behaviour  12.1 Restructuring of physical environment  1.5 Review behaviour goal(s)  1.7 Review outcome goal(s) |
| Concerns about recruitment of dietitians to deliver the programme leading to decreased referrals due to expectation of long wait time or decreased availability | Barrier | Beliefs about Consequences | **Motivation**  reflective | **Education** - educate about ways of enacting the desired behaviour  **Persuasion** – using communication to induce positive or negative feelings or stimulate action  **Modelling** – providing an example for people to aspire to or imitate | 5.3 Information about social and environmental consequences  5.1 Information about health consequences  2.2 Feedback on behaviour  2.7 Feedback on outcome of behaviour  7.1 Prompts/cues  2.3 Self- monitoring of behaviour  9.1 Credible source  5.3 Information about social and environmental consequences  5.1 Information about health consequences  2.2 Feedback on behaviour  2.7 Feedback on outcome(s) of the behaviour  6.1 Demonstration of the behaviour |
| Long waiting time between referral and enrolment into the DPP | Barrier | Environmental context and resources | **Opportunity**  Physical | **Training** – imparting skills  **Environmental restructuring** – changing the physical or social context  **Enablement –** increasing means/ reducing barriers to increase capability (beyond education and training) or opportunity (beyond environmental restructuring) | 6.1 Demonstration of the behaviour  4.1 Instruction on how to perform a behaviour  2.2 Feedback on behaviour  2.7 Feedback on outcome of behaviour  2.3 Self- monitoring of behaviour  8.1 Behavioural practice/ rehearsal  12.5 Adding objects to the environment  7.1 Prompts/ cues  12.1 Restructuring the physical environment  3.1 Social support (unspecified)  3.2 Social support (practical)  1.1 Goal setting (behaviour)  1.3 Goal setting (outcome)  12.5 Adding objects to the environment  1.2 Problem solving  1.4 Action planning  2.3 Self-monitoring of behaviour  12.1 Restructuring of physical environment  1.5 Review behaviour goal(s)  1.7 Review outcome goal(s) |
| Concern about the accessibility and availability of the programme in rural and local areas | Barrier | Knowledge | **Capability**  Psychological | **Education**-  educate about ways of enacting the desired behaviour | 5.3 Information about social and environmental consequences  5.1 Information about health consequences  2.2 Feedback on behaviour  2.7 Feedback on outcome of behaviour  7.1 Prompts/cues  2.3 Self- monitoring of behaviour |
