# Supplementary material for: Designing an implementation strategy to improve referral from general practice to a National Diabetes Prevention Programme using a Delphi survey with healthcare professionals and the Behaviour Change Wheel
Source: BMJ Open. 2026 Feb 16;16(2):e104300. doi: 10.1136/bmjopen-2025-104300 (PMC12911679; doi:10.1136/bmjopen-2025-104300)
Supplement: Supplementary file 2 [file bmjopen-2025-104300.reviewer_comments.pdf]

PEER REVIEW HISTORY

BMJ Open publishes all reviews undertaken for accepted manuscripts. Reviewers are asked to complete a checklist review form and are provided with free text boxes to elaborate on their assessment. These free text comments are reproduced below.

ARTICLE DETAILS

Title (Provisional)

Designing an implementation strategy to improve referral from General Practice to a National Diabetes Prevention Programme using a Delphi Survey with Healthcare Professionals and the Behaviour Change Wheel

Authors

Haseldine, Clair; O'Donoghue, Grainne; Kearney, Patricia M; Riordan, Fiona; Humphreys, Margaret; Kirby, Liz; Mc Hugh, Sheena M

VERSION 1 - REVIEW

**Reviewer**	**1**
**Name**	**Papazafiropoulou, Athanasia**
**Affiliation**	**Tzaneio General Hospital of Piraeus**
**Date**	**20-Jul-2025**
**COI**	**None**

It is an interesting manuscript. Authors succeed to present their data in a clear way adding information to the existing literature. Therefore, I have no corrections to do and the manuscript can be published unaltered.

**Reviewer**	**2**
**Name**	**Roper, Karen L.**
**Affiliation**	**University of Kentucky, Family & Community Medicine**
**Date**	**08-Aug-2025**
**COI**	**None**

Thank you for the opportunity to read and comment on this manuscript. As an American reviewer, I approached this with some hesitation; unsure if the findings would find applicability across healthcare systems. The findings seem quite universal, and in fact may be overly so (the stated goal of prioritization still seems subject to clinic-level pragmatic need). I applaud the use of the Behavior Change Wheel to guide the recommendations and do think this work could be informative to other

health systems that seek to identify first steps in an implementation of Nationals Diabetes Program referral initiative so long as the context-dependent nature of the study is better recognized.

Aside from this, which could be addressed if the authors better consider limitations of the work and especially applicability to different practice settings more thoroughly, my edits are relatively minor. I was very interested in this methodology and glad to have reviewed this well written manuscript.

My recommendations:

Abstract

1. Setting (first sentence) is oddly described. This was an online survey methodology (Qualtrics).
2. Line 38: “invited” should be invitations to participate or similar.
3. Line 49: “Consensus was defined” unclear. I read this as ≥70% agreement on classifying items under each of three categories: not important and two categories created from a combined classification of high or very high importance and low or moderate importance. If this is correct, ad realizing the word limit, the authors might try to better describe this methodology (Methods/Data Analysis section p.12 has the benefit of referring to a source article and does better to clarify)

Introduction

1. I wanted to know more about what is being rolled out in Ireland (line 22, p.6), especially for the international audience. Is the National DPP going to provide virtual opportunities (appears so, from last row in Table 2)? Where will sessions be located (thinking about transportation barriers specifically, but there are certainly other concerns that might apply in other countries but that didn’t make the original (17 or 23? See question below) selected from the literature for inclusion in the Delphi round 1 of the study.

The authors state that item selection was based on relevance to the Irish healthcare context (line 23, p.10) but it would be helpful to know also what did not make the list (e.g., in the U.S., insurance reimbursement would make the list) – if not here, then perhaps in the Discussion as a limitation to broader applicability.

Materials and Methods

1. Please clarify why, of the many studies available, the authors limited their list of factors selected to only two studies (p.10 Survey Development), and why these particular studies were selected.

2. I am confused as to why line 41 (p.10) states there were 17 factors for the first round (consistent with Fig 2) but the Supp. File 1 has 23 items listed. P.15 line 38 (Results) states there 21? I am unnecessarily confused.

3. Please eliminate distracting information.

I don’t see the need for the paragraph on p.12 starting at line 13 on the (9 I believe?) intervention functions that link to policy here in the Methods.

I also don’t see the need for the final sentence on patient and public involvement.

> *Both of the above could be moved to the Discussion if deemed important as a limitation or direction for future research.*

Results/Discussion

Is it possible to report other data that would be relevant to their concerns for implementation?: practice size, characteristics of their communities or patient panel (rural vs. urban)? Comments about barriers and facilitators for the integration may be different depending on their panel characteristics.

Added Limitation suggestions:

I would like to see the authors consider limitations and context-dependent nature of the work more thoroughly. As my comments above suggest, I wonder how much the findings are biased by the original selection criteria of items (use of only 2 studies to generate the formative list and unclear elimination of factors deemed irrelevant a priori). That is, authors should make clear under what health systems or practice types these findings are best applicable to and limits on generalizability, especially in an international context. Also, too little known about the practice characteristics of those who participated (unless, as asked above, more can be provided) to judge how their own panel characteristics influenced their opinions.

**Reviewer**	**3**
**Name**	**Katangwe, Thando**
**Affiliation**	**University of East Anglia, Norwich Clinical Trials Unit**
**Date**	**02-Oct-2025**
**COI**	**None**

Thank you for the opportunity to review this manuscript. I commend the manuscript for addressing an important issue in diabetes prevention and for providing a well-described example of how to develop a theoretically informed implementation strategy in primary care. Overall, the study is considered valuable and well-executed, with suggested improvements mainly focused on clarity, consistency, and transparency.

Abstract

- Page 2, line 37 - Spell out HSE at first use and only abbreviate if going to use again in the abstract
- Page 2, line 45 - Under outcome measure it would be helpful to have a couple of examples of the barriers and facilitators that were ranked.
- Page 3, line 26 – no need for abbreviated EHR if not going to use again in the abstract
- Page 3, line 33 – The BCT’s are “a” starting point

Background

- Page 6 line 31-37 – break this sentence up for ease of reading

Materials and methods

- Page 7, line 3-5 – Clarify the study design which I'd say is an implementation study...aiming to design a theoretically informed strategy to improve....
- Page 8, line 26 – correct Figure number. It currently says Figure 0
- Page 9, line 3 – what waitlist are these? Are they GP waiting lists
- Page 11, line 51-55 – It would be helpful to understand the decision-making process behind mapping previously TDF mapped factors onto the COM-B
- Page 12, line 13-19 – really don't this you need to include much detail about policy categories because you didn't use them
  Page 12, line 31-33 – for transparency how many members of the research team were involved in this discussion what was the purpose of the discussion. If purpose was to select the most appropriate BCT's do you think it would have been useful to include other stakeholders in this discussion? I would recommend completion of a COREQ checklist.

Limitation

Attrition – 38% attrition, any reasons why participants didn't complete the survey

---

VERSION 1 - AUTHOR RESPONSE

Response to Reviewer 1: Dr. Athanasia Papazafiropoulou

Comment	Response
It is an interesting manuscript. Authors succeed to present their data in a clear way adding information to the existing literature. Therefore, I have no corrections to do and the manuscript can be published unaltered.	Dr Athanasia Papazafiropolou,<br>Thank you for taking the time to review the manuscript.

Response to Reviewer 2: Dr Karen Roper

Thank you for the opportunity to read and comment on this manuscript. As an American reviewer, I approached this with some hesitation; unsure if the findings would find applicability across healthcare systems. The findings seem quite universal, and in fact may be overly so (the stated goal of prioritization still seems subject to clinic-level pragmatic need). I applaud the use of the Behavior Change Wheel to guide the recommendations and do think this work could be informative to other health systems that seek to identify first steps in an implementation of Nationals Diabetes Program referral initiative so long as the context-dependent nature of the study is better recognized.
Aside from this, which could be addressed if the authors better consider limitations of the work and especially applicability to different practice settings more thoroughly, my edits are relatively minor. I was very interested in this methodology and glad to have reviewed this well written manuscript.

Section	Comment	Response

**Abstract**	**1.Setting** (first sentence) is oddly described. This was an online survey methodology (Qualtrics).<br><br>**2. Line 38:** "invited" should be invitations to participate or similar.<br><br>**3. Line 49:** "Consensus was defined" unclear. I read this as ≥70% agreement on classifying items under each of three categories: not important and two categories created from a combined classification of high or very high importance and low or moderate importance. If this is correct, ad realizing the word limit, the authors might try to better describe this methodology (Methods/Data Analysis section p.12 has the benefit of referring to a source article and does better to clarify)	1. The first sentence under setting has been revised: Page 2, line 12<br><br>"The survey was conducted online with healthcare professionals working in the primary care setting in Ireland (April to June 2024)."<br><br>2. This sentence has been corrected: page 2 line 19 - 21<br><br>"Recruitment was through a number of gatekeepers, Health Service Executive manager and professional groups who shared invitations to participate with eligible healthcare professionals."<br><br>3. The methodology is described in more detail:<br>Page 3 lines 2 - 9<br><br>"Consensus was defined as agreement of ≥70% for each factor, in the combined categories of high importance/very high importance, low/moderate importance or not important. Factors not reaching consensus after the first round were included in Round 2 with any new factors from Round 1. Factors that did not reach consensus or reached consensus as not important or low/moderate importance were excluded. Only factors reaching consensus as being high importance/very high importance across the two rounds were included in the final prioritised list."
**Introduction**	**1.** I wanted to know more about what is being rolled out in Ireland (line 22, p.6), especially for the international audience. Is the National DPP going to provide virtual opportunities (appears so, from last row in Table 2)?<br>Where will sessions be located (thinking about transportation barriers specifically, but there are certainly other concerns that might apply in other countries but that didn't make the original (17 or 23? See question below) selected from the literature for inclusion in the Delphi round 1 of the study.	1. More information has been added about the roll out of the NDPP:<br>Page 7 lines 1-5<br><br>"The health service in Ireland is implementing a National Diabetes Prevention Programme (NDPP). The programme was piloted in 2021/22 as an online programme and it is currently being rolled out nationally on a phased basis, with face-to-face delivery (since mid-2024) in addition to online delivery. The NDPP is accessed via GP referral to

		community specialist teams outside of the hospital setting."
	The authors state that item selection was based on relevance to the Irish healthcare context (line 23, p.10) but it would be helpful to know also what did not make the list (e.g., in the U.S., insurance reimbursement would make the list) – if not here, then perhaps in the Discussion as a limitation to broader applicability.	The list of factors from the systematic review, which informed the survey, has now been included as supplementary file 2 and the factors not included are specified.<br><br>Page 10 lines 7-10<br><br>"A list of factors affecting referral was generated from a previous qualitative study with attenders and educators of the NDPP (14) and a systematic review of 37 studies on barriers and facilitators to referral from healthcare workers' perspectives (5) (Supplementary Files 1,2)."<br><br>Supplementary File 2 (See below)<br><br>Page 10 lines 20-21<br><br>"Factors relating to insurance cover were not included as there is no charge to attend the NDPP in Ireland."
**Materials and methods**	1. Please clarify why, of the many studies available, the authors limited their list of factors selected to only two studies (p.10 Survey Development), and why these particular studies were selected.	1.These two studies reflect the first stage of Behaviour Change Wheel framework namely *understanding the behaviour* and they sought to understand factors specific to the Irish context (14) and broader international evidence (5). As such the list of items reflects broader contextual factors that were relevant. These two papers refer to the research preceding and informing the final stages of the Behaviour Change Wheel in this study. The two papers include a qualitative study with attenders and educators in the NDPP pilot (the only research on an NDPP in Ireland at that time) (Haseldine et al, 2024) and a systematic review of 37 papers on perceptions of HCWs on referral to DPPs (Haseldine et al, 2025).

		The following sentence was added to ***Survey development*** to clarify the choice of the two articles:<br><br>Page 10 lines 10-16<br><br>"These studies informed the list as the qualitative study (14) was the only published article on a NDPP in Ireland at that time and the systematic review was a comprehensive analysis of the international literature on perspectives on referral to DPPs (5). These two studies reflect the first stage of Behaviour Change Wheel framework namely *understanding the behaviour* and they sought to understand factors specific to the Irish context (14) and broader international evidence (5). As such the list of items reflects broader contextual factors that were relevant."<br><br>References:<br><br>5. Haseldine C, Pallin JA, Kearney PM, Mc Hugh SM, Riordan F, Cotterill S, et al. Healthcare workers' perspectives on barriers and facilitators to referral to type 2 diabetes prevention programmes: a systematic review. BMJ Open [Internet]. 2025 Mar 1;15(3):e090105.<br><br>14. Haseldine C, O'Donoghue G, Kearney PM, Riordan F, Kerins C, Kirby L, et al. Factors influencing participation in an online national diabetes prevention programme: A qualitative study with attenders and educators. Diabet Med. 2024 Jun;41(6):e15277.
	2. I am confused as to why line 41 (p.10) states there were 17 factors for the first round (consistent with Fig 2) but the Supp. File 1 has 23 items listed. P.15 line 38 (Results) states there 21? I am unnecessarily confused.	2. The supplementary file listed the survey ***question numbers*** 7 – 23 (17 items). I have added an item number to the list to avoid confusion (see Supplementary File 1 below).<br><br>There were 4 new suggestions from the first round of the Delphi. This gives the

	3. Please eliminate distracting information.<br>I don't see the need for the paragraph on p.12 starting at line 13 on the (9 I believe?) intervention functions that link to policy here in the Methods.<br><br>I also don't see the need for the final sentence on patient and public involvement.<br>Both of the above could be moved to the Discussion if deemed important as a limitation or direction for future research	total of 21 factors. I have clarified this under ***Results: Delphi Round 2***<br>Page 15 lines 26-29<br><br>"From the two rounds of 21 factors (17 factors from Round 1 and 4 new factors added in Round 2), consensus was achieved on 12 factors (barriers and facilitators) being of high/very high importance to address in a strategy to improve NDPP referral."<br><br>3. The paragraph on interventions functions linking to policy categories has been removed.<br>Page 12 lines 20-25<br><br>BMJ Open requires authors to include a Patient and Public Involvement statement in a subsection within the methods of their papers therefore we have retained this sentence.
**Results/Discussion**	Is it possible to report other data that would be relevant to their concerns for implementation?: practice size, characteristics of their communities or patient panel (rural vs. urban)? Comments about barriers and facilitators for the integration may be different depending on their panel characteristics.	No data were collected on practice setting in this study as the focus was to gain consensus and prioritise factors affecting referral. The role of practice characteristics would be important to consider in further research on the implementation of the NDPP. I have acknowledged this limitation in the S***trengths and limitations*** section:<br><br>Page 23 lines 18-28<br><br>"One final limitation is that data on the practice settings of the Delphi respondents were not collected. The characteristics of the practice (e.g., rural vs urban setting, practice size, characteristics of the communities) may have influenced perspectives on the barriers and facilitators faced. A 2025 systematic review of healthcare workers perceptions on referral to DPPs found referral more difficult in more rural, less populated areas or areas with poor transportation [5]. A qualitative study on clinicians' perspectives of diabetes prevention, reported that the high

		prevalence of diabetes in a resource constrained safety net health care setting created an expectation to prioritise the treatment of diabetes over prevention [35]. Future research could explore the influence of contextual factors on healthcare professionals' perspectives to identify modifiable factors in different healthcare settings ."<br><br>References:<br><br>5. Haseldine C, Pallin JA, Kearney PM, Mc Hugh SM, Riordan F, Cotterill S, et al. Healthcare workers' perspectives on barriers and facilitators to referral to type 2 diabetes prevention programmes: a systematic review. BMJ Open [Internet]. 2025 Mar 1;15(3):e090105.<br><br>14. Haseldine C, O'Donoghue G, Kearney PM, Riordan F, Kerins C, Kirby L, et al. Factors influencing participation in an online national diabetes prevention programme: A qualitative study with attenders and educators. Diabet Med. 2024 Jun;41(6):e15277.<br><br>35. Gore R, Brown A, Wong G, Sherman S, Schwartz M, Islam N. Integrating community health workers into safety-net primary care for diabetes prevention: qualitative analysis of clinicians' perspectives. Journal of general internal medicine. 2020 Apr;35(4):1199-210.
**Added Limitation suggestions:**	I would like to see the authors consider limitations and context-dependent nature of the work more thoroughly. As my comments above suggest, I wonder how much the findings are biased by the original selection criteria of items (use of only 2 studies to generate the formative list and unclear elimination of factors deemed irrelevant a priori).	To elaborate on the context of this study we have added the following information and addressed concerns about the selection criteria for items in the following sections:<br><br>In the **Materials and methods**, as outlined in the section above, we have clarified that the two studies that informed the list reflected the first stage of the Behaviour Change Wheel and sought to understand factors specific to the Irish context and broader international evidence.

		Acknowledging that some factors may not be relevant to the Irish context but could speak to the transferability of findings, we have included the full list in supplementary file 2.<br><br>All of the factors affecting referral were included in the Delphi study except for those relating to insurance cover for the programme as the programme is free to access in Ireland. This has been noted under *Survey development*:<br>Page 10 lines 20-21<br><br>"Factors relating to insurance cover were not included as there is no charge to attend the NDPP in Ireland."
	That is, authors should make clear under what health systems or practice types these findings are best applicable to and limits on generalizability, especially in an international context.	In the ***Strengths and limitations,*** I have outlined the generalisability of the results:<br>Page 22 lines 12-31<br>Page 23 lines 1-3<br><br>"The original list of factors affecting referral, that were prioritised in the Delphi, were drawn from a systematic review of 37 studies conducted in the US, England and Australia (5) and a qualitative study from Ireland (14) which are all higher income countries. These findings could be applied to private (US) public (UK) or mixed public and private healthcare systems (Ireland and Australia). The importance of the referral pathway (clear and easy pathway, electronic referral), healthcare professional workload and resources (time and staff) and concern about the burden of attending (length of time involved) were important factors across all systems. Concern about the financial cost of the programme and insurance cover was an important factor in the US (5). The factors from the original list prioritised by Irish healthcare professionals in in the Delphi survey are specific to the Irish primary care context at the time of the survey, however they may be applicable to other healthcare

		systems. In Ireland the majority of the population pay to access services in primary care including General Practitioners (GPs) who are gatekeepers for a range of primary and secondary care services (34). An ageing population and the development of new technologies and treatments are increasing the demand for services, and this, coupled with a healthcare workforce shortage, can result in long waiting times to access care (34). Reforms in the Irish healthcare service are aiming to increase care in the community with chronic disease management and prevention programmes accessed through GPs, including the National Diabetes Prevention Programme. While contextual to Ireland, the factors prioritised in the Delphi survey could therefore be generalisable to health systems rolling out DPPs that have healthcare workforce shortages, mixed public and private healthcare systems and systems experiencing increasing demand for services."<br><br>References:<br><br>5. Haseldine C, Pallin JA, Kearney PM, Mc Hugh SM, Riordan F, Cotterill S, et al. Healthcare workers' perspectives on barriers and facilitators to referral to type 2 diabetes prevention programmes: a systematic review. BMJ Open [Internet]. 2025 Mar 1;15(3):e090105.<br><br>14. Haseldine C, O'Donoghue G, Kearney PM, Riordan F, Kerins C, Kirby L, et al. Factors influencing participation in an online national diabetes prevention programme: A qualitative study with attenders and educators. Diabet Med. 2024 Jun;41(6):e15277.<br><br>34. Connolly S, Brick A, O'Neill CJ, O'Callaghan M. An analysis of the primary care systems of Ireland and Northern Ireland. Research Series; 2022.

	Also, too little known about the practice characteristics of those who participated (unless, as asked above, more can be provided) to judge how their own panel characteristics influenced their opinions.	I have acknowledged this in the S***trengths and limitations*** section:<br><br>Page 23 lines 18-28<br><br>"One final limitation is that data on the practice settings of the Delphi respondents were not collected. The characteristics of the practice (e.g., rural vs urban setting, practice size, characteristics of the communities) may have influenced perspectives on the barriers and facilitators faced. A 2025 systematic review of healthcare workers perceptions on referral to DPPs found referral more difficult in more rural, less populated areas or areas with poor transportation (5). A qualitative study on clinicians' perspectives of diabetes prevention, reported that the high prevalence of diabetes in a resource constrained safety net health care setting created an expectation to prioritise the treatment of diabetes over prevention (35). Future research could explore the influence of contextual factors on healthcare professionals' perspectives to identify modifiable factors to improve referral to DPPs in different healthcare settings."<br><br>References<br><br>5. Haseldine C, Pallin JA, Kearney PM, Mc Hugh SM, Riordan F, Cotterill S, et al. Healthcare workers' perspectives on barriers and facilitators to referral to type 2 diabetes prevention programmes: a systematic review. BMJ Open [Internet]. 2025 Mar 1;15(3):e090105.<br><br>35. Gore R, Brown A, Wong G, Sherman S, Schwartz M, Islam N. Integrating community health workers into safety-net primary care for diabetes prevention: qualitative analysis of clinicians' perspectives. Journal of general internal medicine. 2020 Apr;35(4):1199-210.

Response to Reviewer 3: Dr Thando Katangwe

Thank you for the opportunity to review this manuscript. I commend the manuscript for addressing an important issue in diabetes prevention and for providing a welldescribed example of how to develop a theoretically informed implementation strategy in primary care. Overall, the study is considered valuable and well-executed, with suggested improvements mainly focused on clarity, consistency, and transparency.

Section	Comment	Response
**Abstract**	**Page 2, line 37** - Spell out HSE at first use and only abbreviate if going to use again in the abstract	The abbreviation HSE has been spelled out;<br>Page 2 line 19<br>"Recruitment was through a number of gatekeepers, Health Service Executive manager and professional groups who shared invitations to participate with eligible healthcare professionals."
	**Page 2, line 45** - Under outcome measure it would be helpful to have a couple of examples of the barriers and facilitators that were ranked.	The following examples of barriers and facilitators were added to the O**utcome measure** section:<br>Page 2 lines 26 – 29<br>Page 3 line 1<br><br>"Barriers included limited practical information about the availability of the programme, concerns about workload, competing priorities and concern about patient motivation, the time commitment for patients and referral delays. Facilitators included electronic referral and feedback, promotion of the programme by healthcare professionals and consultation with patients before referral."
	**Page 3, line 26** – no need for abbreviated EHR if not going to use again in the abstract	The abbreviation EHR has been removed:<br>Page 3 line 21<br><br>"The health service should continue to provide electronic referrals and electronic prompts to refer could be considered as part of the electronic health record."
	**Page 3, line 33** – The BCT's are "a" starting point	This sentence has been corrected with "a" added:<br>Page 3 line 25<br><br>"The BCTs are a starting point for a strategy to improve referral to DPPs."

**Background**	**Page 6 line 31-37** – break this sentence up for ease of reading	The sentence has been broken up.<br>Page 7 lines 16-20<br><br>"This study aims to gain consensus on and prioritise the most important factors influencing referral to a NDPP among healthcare professionals involved in referral. The study also aims to use the BCW to select behaviour change techniques (BCTs) for an implementation strategy to improve referral to the programme in the future."
**Materials and methods**	**Page 7, line 3-5** – Clarify the study design which I'd say is an implementation study…aiming to design a theoretically informed strategy to improve….	The study design has been clarified.<br>Page 7 lines 26-27<br><br>"This implementation study aiming to develop a theoretically informed strategy to improve referral is broadly based on the Behaviour Change Wheel (BCW) (16)."
	**Page 8, line 26** – correct Figure number. It currently says Figure 0	Figure 1 corrected<br>Page 8 line 16
	**Page 9, line 3** – what waitlist are these? Are they GP waiting lists	The source of the waiting list has been clarified:<br>Page 9 line 3-5<br><br>"During the pilot, participants eligible for the NDPP were identified through existing waiting lists for primary care dietitians and by referral from GPs who had been informed of the programmes in their areas."
	**Page 11, line 51-55** – It would be helpful to understand the decision-making process behind mapping previously TDF mapped factors onto the COM-B	This information has been added:<br>Page 12 lines 9-13<br><br>"The mapping process followed the guidance in the Behaviour Change Wheel manual (16). The TDF gives a detailed understanding of the specific factors influencing referral behaviour and the COM B is a higher-level classification which facilitates the mapping of these factors to intervention functions to change that behaviour (Supplementary File 3)."<br><br>Supplementary file 3 which illustrates the process has also been added (see Supplementary File 3 below).

	**Page 12, line 13-19** – really don't this you need to include much detail<br>about policy categories because you didn't use them	In response to this comment and that of Reviewer 2, the paragraph on policy categories has been deleted.<br>Page 12 lines 20-25
	**Page 12, line 31-33** – for transparency how many members of the research team were involved in this discussion what was the purpose of the discussion.	The initials and further information on the five members of the research team involved in this discussion have been added:<br>Page 12 lines 29-30<br>Page 13 lines 6-11<br>Supplementary File 4 – see below<br><br>"The research team (CH, GO'D, PK, FR, SMcH) discussed the BCT shortlist in an online meeting (lasting 90 minutes) by considering how the BCT could be operationalised within the context of referral to the NDPP."<br><br>"The five members of the research team involved in this discussion were female academics, four of which were healthcare professionals. The lead author (CH) was a PhD scholar/healthcare professional with an interest in chronic disease prevention and experience delivering a diabetes prevention programme. The results of the meeting were summarised by the lead author (CH) with the final list of BCTs returned to the research team to check for accuracy (See COREQ checklist- Supplementary File 4). "
	If purpose was to select the most appropriate BCT's do you think it would have been useful to include other stakeholders in this discussion?	The purpose of the discussion was to choose the most appropriate BCTs. A range of stakeholders were involved to bring the research to this stage (NDPP attenders, NDPP educators, NDPP management, GPs, practice nurses). Choosing the BCTs was carried out by the research team (which included healthcare professional representation) as we believed this stage could be challenging due to the number of BCTs to consider, technical language used in the taxonomy and the need for some familiarity with the BCW and BCT Taxonomy. The rationale for this approach has been added:<br>Page 13 lines 1-5<br><br>"Different approaches are used for selection of BCTs, sometimes led by researchers and other

	I would recommend completion of a COREQ checklist.	times involving health service stakeholders (32, 30). We opted for research-led approach to minimise the burden on HCPs who were already heavily involved in the qualitative study to *understand the behaviour* (BCW Stage 1) and the Delphi survey."<br><br>The next step in this process to operationalise the strategy would be further consultation with stakeholders to discuss the acceptability, feasibility and modes of delivery in the Irish context which is acknowledged in the discussion page 21 lines 28-30.<br><br>A COREQ checklist has been completed and is included as Supplementary File 4 (See below).
**Limitation**	Attrition – 38% attrition, any reasons why participants didn't complete the survey	Every effort was made to increase response rates to Round 2 (limited response time to 10 minutes, short time between rounds, limiting the number of rounds and sending reminders). We did not follow up with participants who did not complete Round 2 and therefore do not have any information for on their reasons for dropping out.

VERSION 2 - REVIEW

**Reviewer**	**3**
**Name**	**Katangwe, Thando**
**Affiliation**	**University of East Anglia, Norwich Clinical Trials Unit**
**Date**	**27-Nov-2025**
**COI**	

No comments. Well done for addressing all reviewers comments.
